# Time-course changes in energy expenditure in sepsis: a prospective observational study

**DOI:** 10.1186/s13613-025-01592-3

**Published:** 2025-10-14

**Authors:** Weronika Wasyluk, Robert Fiut, Izabela Świetlicka, Magdalena Szukała, Agnieszka Zwolak, Joop Jonckheer, Wojciech Dąbrowski

**Affiliations:** 1 Department of Internal Medicine and Internal Nursing, Medical University of Lublin, ul. Chodźki 7, Lublin, 20-093 Poland; 2 Department of Clinical Physiotherapy, Medical University of Lublin, Lublin, Poland; 3 Department of Biophysics, University of Life Sciences in Lublin, Lublin, Poland; 4 Clinical Intensive Care Unit, University Clinical Hospital, No. 4 in Lublin, Lublin, Poland; 5 Department of Intensive Care Medicine, Universitaire Ziekenhuis Brussel (UZ Brussel), Vrije Universiteit Brussel (VUB), Brussels, Belgium; 6 1st Department of Anaesthesiology and Intensive Therapy, Medical University of Lublin, Lublin, Poland

**Keywords:** Sepsis, Metabolism, Respiratory quotient, ICU, Indirect calorimetry

## Abstract

**Background:**

Sepsis is associated with dynamic metabolic alterations influencing energy expenditure and substrate utilization. This study aimed to evaluate time-course changes in energy metabolism in critically ill patients with sepsis and identify clinical and nutritional predictors of resting energy expenditure (REE) and respiratory quotient (RQ).

**Methods:**

In this prospective observational study, 30 mechanically ventilated adult patients with sepsis were assessed using indirect calorimetry on days 1, 2, 3, 5, and 7 following diagnosis. Nutritional treatment, biochemical markers, and clinical variables were recorded. Linear mixed-effects models were applied to evaluate temporal changes and identify predictors of REE and RQ.

**Results:**

REE increased by Day 5 versus Day 1 (+ 163.7 kcal/day; *p* = 0.049), with a concurrent rise in RQ (*p* = 0.013). Higher body temperature, higher arterial pH, a greater protein-to-non-protein calorie ratio, and higher protein intake were associated with higher REE, whereas higher lactate concentrations and use of CRRT were associated with lower REE. RQ was positively associated with energy intake, REE coverage, and blood glucose. Clinical-severity scores and inflammatory markers showed no significant associations with REE or RQ.

**Conclusions:**

Energy metabolism in sepsis evolves dynamically, with significant changes in REE and substrate utilization over time. Temperature, acid-base balance, CRRT, and nutritional strategies were associated with variability in energy expenditure. These findings support the need for individualised metabolic assessment and targeted nutritional strategies in critically ill patients with sepsis.

**Graphical abstract:**

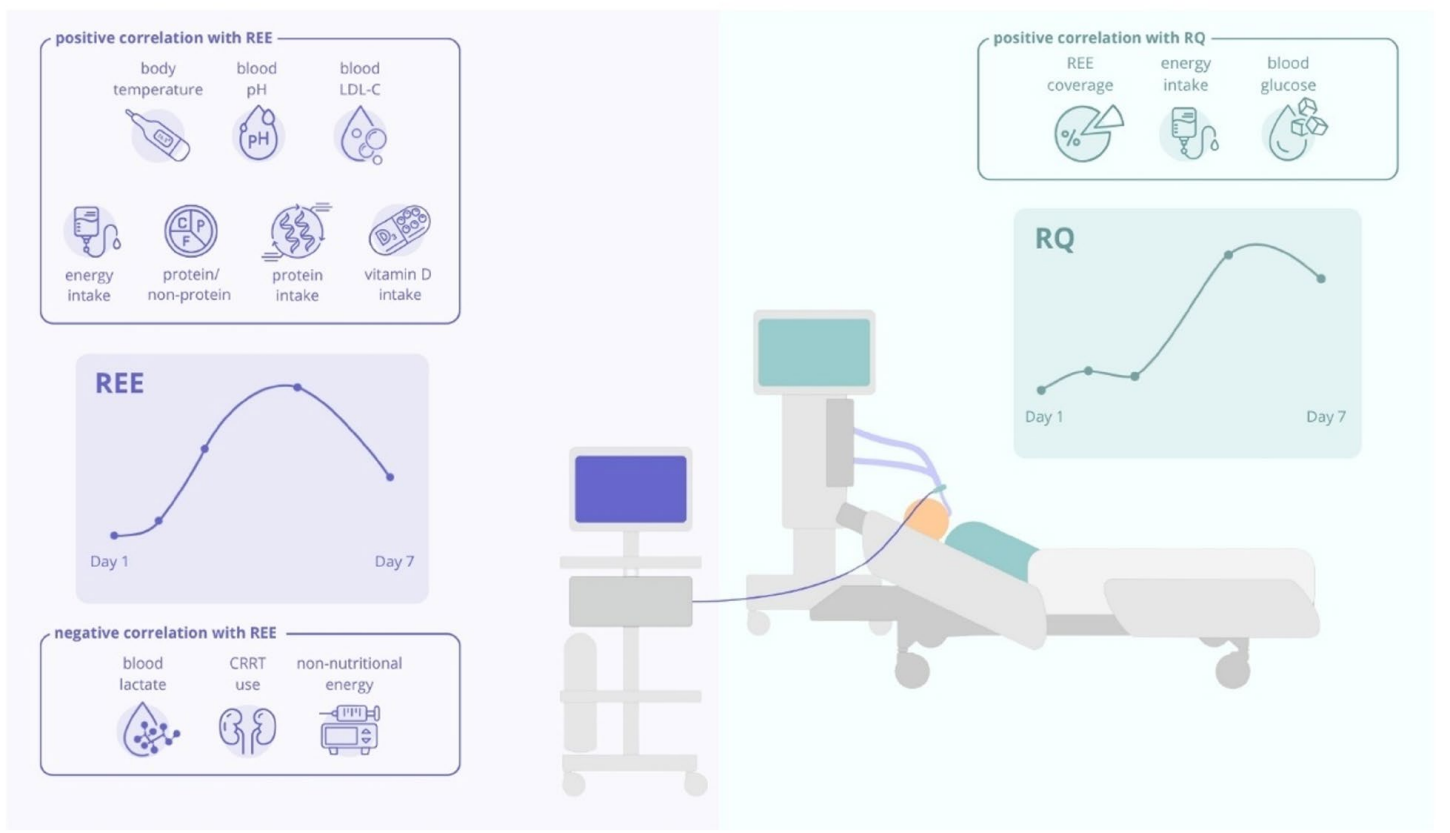

**Supplementary Information:**

The online version contains supplementary material available at 10.1186/s13613-025-01592-3.

## Background

Sepsis is defined as life-threatening organ dysfunction caused by dysregulated host systemic inflammatory and immune response to infection [1]. Endocrine and metabolic disturbances, including dysregulation of carbohydrate, protein, and lipid metabolism, are frequently observed in patients with sepsis [2, 3]. These disturbances are closely linked to mitochondrial dysfunction, which is critical in the cellular energy imbalance observed in patients with sepsis [4]. Impaired mitochondrial function leads to reduced energy production and exacerbates the body’s inability to efficiently utilize key metabolic substrates [5, 6].

Both metabolic and endocrine disturbances in sepsis exhibit a dynamic, phased nature. During the acute phase, the body experiences a hypermetabolic response, marked by an uncontrolled inflammatory response, elevated energy expenditure, and enhanced catabolism. This phase is driven by heightened neuroendocrine activity, with increased secretion of catecholamines, cortisol, and inflammatory cytokines [7–9]. As the condition progresses, many patients enter a hypometabolic phase, characterised by a downregulation of metabolic activity, possibly as an adaptive mechanism to conserve energy and prevent further cellular damage [10–13].

Understanding the dynamic nature of metabolic and endocrine disturbances in sepsis is essential for effectively managing critically ill patients. Accurate assessment of energy requirements is crucial, as insufficient and excessive energy provision may negatively impact clinical outcomes [14]. Indirect calorimetry (IC) is the recommended method for measuring energy expenditure in this population, enabling tailored nutritional interventions [15, 16]. However, most studies in this area are limited to isolated measurements, without capturing the temporal evolution of metabolic changes. The influence of the duration and severity of sepsis and therapeutic and nutritional interventions on energy metabolism remains insufficiently explored. A more comprehensive, time-based approach is necessary to understand how energy expenditure and substrate utilization evolve during sepsis. This knowledge can help optimise patient management and improve survival rates.

To address these gaps, this prospective observational study aimed to evaluate the temporal dynamics of metabolism in critically ill patients with sepsis. A secondary objective was to assess the association between selected clinical and nutritional factors and changes in energy metabolism over time.

## Methods

This prospective, observational, single-centre study was conducted in accordance with the Declaration of Helsinki and the guidelines of the Bioethics Committee of the Medical University of Lublin (approval number KE-0254/323/2019). The study was conducted between January 2021 and December 2023 in the 1st Clinic of Intensive Therapy at the Medical University of Lublin, Poland, in accordance with the local intensive care unit (ICU) protocol and adhering to the STROBE guidelines [17]. Informed consent was obtained from the legally authorised representative or closest family member in cases where the patient was unable to provide consent. When the patient’s condition subsequently allowed, the consent was reaffirmed by the patient. As this was an observational study, the attending physician determined all interventions, including the administration of nutritional and non-nutritional energy sources. The study population included adult patients (age >18) treated for sepsis (according to the Sepsis-3 criteria) with mechanical ventilation due to sepsis-related respiratory insufficiency. Patients were enrolled at the time of sepsis diagnosis according to the Sepsis-3 criteria, regardless of whether sepsis was the reason for ICU admission or developed during the ICU stay for another cause. Infection was suspected based on clinical signs and symptoms, laboratory abnormalities, and/or radiological findings, and confirmed by positive microbiological results. In all patients with suspected infection, blood, urine, and bronchoalveolar lavage (BAL) fluid were collected for microbiological examination. Additionally, samples for microbiological testing were obtained from each site clinically suspected of infection. Exclusion criteria included pregnancy and factors that could compromise the reliability or validity of IC measurements (fraction of inspired oxygen (FiO₂) >70%, positive end-expiratory pressure (PEEP) >12 cm H₂O, chest drainage, or the presence of gases other than oxygen (O₂), carbon dioxide (CO₂), and nitrogen (N₂) in the breathing mixture), non-steady-state ventilatory conditions or cases in which the exact onset of sepsis could not be determined. These eligibility criteria were applied throughout the entire study period; if a patient ceased to meet them at any point during follow-up, their participation in the study was terminated. Measurements were performed on Days 1 (baseline), 2, 3, 5, and 7 following sepsis diagnosis. If a patient no longer fulfilled the eligibility criteria or died during the observation period, their participation in the study was concluded on the day of their last valid measurement. During the observation, the following data were recorded: actual body weight (ABW) and temperature, body mass index (BMI), information on the use of continuous renal replacement therapy (CRRT), parameters measured by IC, assessment of the patient’s condition according to selected scales, inflammatory parameters, metabolic parameters, energy and nutrient supply (energy intake, protein-to-non-protein calorie ratio, non-nutritional energy share, resting energy expenditure (REE) coverage, protein intake per kilogram of ABW, protein intake per kilogram of ideal body weight (IBW) and intake of vitamins). Definitions and calculation methods of the above variables are provided in Table S1 in the Supplementary Materials.

### Clinical scoring systems

Severity of sepsis was evaluated using the Acute Physiology Score (APS) [18] and the Sequential Organ Failure Assessment (SOFA) [19]. Because patients were sedated, the Glasgow Coma Scale was omitted from these scoring systems. The level of sedation was assessed with the Richmond Agitation-Sedation Scale (RASS) [20], and nutritional risk was evaluated using the Nutrition Risk in Critically Ill (NUTRIC) score [21].

### Indirect calorimetry

REE was measured with a COSMED Quark RMR calorimeter (COSMED, Rome, Italy) in accordance with the “Checkpoints for successful indirect calorimetry” published by Oshima et al. [22]. A minimum recording of 30 min was obtained, and data quality was verified using predefined steady-state criteria. The collected parameters included oxygen consumption (V̇O₂) [ml/min] and carbon dioxide production (V̇CO₂) [ml/min]. REE was calculated using the Weir equation, and the respiratory quotient (RQ) was determined as V̇CO₂/V̇O₂. In patients receiving CRRT, CO₂ loss via the circuit was corrected using the method previously described by our group (23).

### Data and sample collection

Demographic data (age, sex, race/ethnicity, height) were abstracted from the medical record. ABW was recorded on the study day using the integrated bed scale. Information on medications – including nutrition therapy – was extracted from the physician orders and medication administration record. Composition of nutritional and non-nutritional energy/nutrient sources was obtained from the Summary of Product Characteristics (SmPC); when SmPCs lacked detail or were unavailable, data were retrieved from the manufacturer’s website or obtained directly from the manufacturer via email. Daily energy intake, protein, carbohydrates, fats, and vitamins were calculated. IBW was defined as the weight corresponding to a BMI of 25 kg/m² and calculated for all patients to provide a standardised, height-based denominator for protein-per-kg analyses.

Arterial blood samples were drawn aseptically from the indwelling arterial line ≥ 15 min after the start of the IC recording using dedicated tubes (Blood Gas, S-Monovette^®^; Serum CAT, S-Monovette^®^; K3-EDTA, S-Monovette^®^). Arterial blood gas analysis (pH, sodium (Na⁺), potassium (K⁺), partial pressure of carbon dioxide (pCO₂), partial pressure of oxygen (pO₂), glucose, lactate) was performed immediately on a GEM^®^ Premier 5000 analyzer. Routine laboratory testing included creatinine, bilirubin, C-reactive protein (CRP), procalcitonin (PCT), interleukin-6 (IL-6), white blood cell count (WBC), platelet count (PLT), hematocrit (HCT), hemoglobin (Hb), total cholesterol (TC), low-density lipoprotein cholesterol (LDL-C), high-density lipoprotein cholesterol (HDL-C), triglycerides, and albumin.

### Statistical analysis

Owing to patient deaths, ICU discharge, and technical limitations of indirect calorimetry, the dataset was unbalanced. Traditional repeated-measures procedures assume complete data and missing completely at random (MCAR), which is unrealistic in this setting. We therefore applied Generalised Estimating Equations (GEE) and Linear Mixed-Effects Models (LMM), which accommodate incomplete longitudinal data under the missing at random (MAR) assumption. As missingness was largely related to observed baseline severity included in the models, we considered MAR a reasonable working framework.

GEE were applied to evaluate changes in clinical variables over time, modelling time categorically (reference: Day 1). This design estimates population-averaged, day-by-day changes (vs. Day 1) while accounting for within-patient correlation and intermittent missingness. Estimated marginal means (EMMs) with Bonferroni adjustment supported pairwise contrasts across time points.

LMMs used a random intercept for patient and an autoregressive model of order 1 (AR (1)) residual structure to model temporal changes in REE and RQ. Fixed effects (time, sex and age) were selected a priori for their physiological relevance to REE. A base model (age, sex) was fit first; candidate time-varying clinical and nutritional predictors were then added one at a time to estimate their independent associations while adjusting for baseline covariates. Clinical and nutritional predictors were prespecified based on clinical plausibility, relevance to energy metabolism, and availability across time points. The resulting complete model included the fixed effects time, age, sex and the predictor of interest, plus a random intercept for patient and an AR (1) autocorrelation structure. Standard errors (SE) and p-values are based on restricted maximum likelihood estimation (REML).

Missing data were assumed MAR. No imputation was performed; LMMs used all available observations under likelihood, and GEE provided population-averaged estimates.

Model selection contrasted additive versus time-by-predictor interaction forms, each with and without AR (1), using the Akaike information criterion (AIC) and Bayesian information criterion (BIC), favouring lower values. Multicollinearity was assessed via variance inflation factors (VIF) – all VIF < 5. Model diagnostics included residual plots, Shapiro–Wilk tests for approximate normality, and autocorrelation-function (ACF) checks; marginal and conditional R² (performance package) summarised explained variance. Fixed-effect significance is reported with two-sided p-values; multiplicity for planned pairwise contrasts was controlled by Bonferroni adjustment. For LMMs of REE and RQ, a post-hoc power check was based on EMM contrasts for the largest observed time-point differences.

Continuous data are presented as mean ± SE or median [interquartile range, IQR], as appropriate. Analyses were performed in Statistica 13.3 (TIBCO Software, Palo Alto, CA, USA), RStudio (2024.12.0 Build 467, Posit Software, PBC, Boston, MA URL http://www.rstudio.com/) using the following packages: nlme, lme4, car, emmeans, performance, ggeffects, ggpredict, lmerTest, DHARMa, ggplot2, G*Power 3.1 (Heinrich-Heine-Universität Düsseldorf, Germany) and OriginPro 2022b v.9.95 (OriginLab, Northampton, MA, USA).

## Results

### General characteristics

Thirty Caucasian adult patients were studied: 10 women (33.3%) and 20 men (66.6%). Of the 30 patients, 26 (87%) had a BMI > 25 kg/m² at the beginning of observation (Day 1). No patients were underweight (BMI < 18.5 kg/m²). At inclusion, patients had a median temperature of 36.6 °C [35.9–37.5], with 20% exhibiting fever. Baseline characteristics are presented in Table 1.

**Table 1:** Baseline demographic, metabolic and inflammatory characteristics of the patients on the first day of observation (Day 1)

Variable	*N*	Median	IQR	Min-Max
Age (years)	30	66.0	55.0–75.0	29.0–89.0
Height (cm)	30	170.0	165.0-175.0	146.0-185.0
IBW (kg)	30	72.5	68.1–76.9	51.5–85.6
ABW (kg)	30	85.5	73.0-99.5	57.5–124.0
BMI (kg/m^2^)	30	30.7	26.0-33.8	20.7–46.6
Temperature (°C)	30	36.6	35.9–37.5	33.7–40.0
Albumin (g/dl)	28	2.92	2.83–3.11	2.19–3.34
TC (mg/dl)	28	94.0	78.5–112.0	45.0-186.0
LDL-C (mg/dl)	28	41.5	34.8–56.2	15.0-119.0
HDL-C (mg/dl)	28	13.5	7.8–16.8	5.0–41.0
Triglycerides (mg/dl)	29	171.0	131.0-208.0	71.0-434.0
Glucose (mg/dl)	30	137.0	121.8–164.0	60.0-264.0
Lactate (mmol/l)	30	1.2	0.8–1.6	0.3–13.1
Arterial pH	30	7.39	7.28–7.44	7.11–7.59
CRP (mg/l)	27	248.4	182.0-318.1	56.7-505.6
PCT (ng/ml)	30	12.61	6.30–20.00	0.26–515.2
IL-6 (pg/ml)	27	201.3	147.0-315.6	7.6–5000
WBC (10^3^/µl)	30	14.3	9.2–22.1	4.8–49.0
SOFA	30	8.0	6.0-10.8	4.0–14.0
APS	30	7.0	4.0–12.0	2.0–18.0
Time from ICU admission to study inclusion (days)	30	4.00	2.00–6.00	1.0–40.0
Time from hospital admission to study inclusion (days)	30	9.00	2.00–25.00	1.0-107.00
Primary reason for ICU admission	10	medical		
	11	surgical		
	5	trauma		
	4	neurological		
Nutrition	17	enteral		
	8	parenteral		
	5	mixed		

Abbreviations: N – number of observations; IQR – interquartile range; Min–Max – minimum–maximum; IBW – ideal body weight; ABW – actual body weight; BMI – body mass index; TC – total cholesterol; LDL-C – low-density lipoprotein cholesterol; HDL-C – high-density lipoprotein cholesterol; CRP – C-reactive protein; PCT – procalcitonin; IL-6 – interleukin-6; WBC – white blood cell count; SOFA – Sequential Organ Failure Assessment; APS – Acute Physiology Score; ICU – intensive care unit

During the ICU stay, 17 patients died (13 within the study window), and the median ICU length of stay was 20.5 days [12.0–41.0], with most patients admitted to the ICU for reasons other than sepsis. Patient flow across study time points, including reasons for study discontinuation, is shown in the Supplementary Materials (Figure S1).

Sepsis was most frequently secondary to pneumonia (20 cases), followed by urinary tract infections [14], infections related to trauma or its complications [4], gastrointestinal perforations [4], and necrotic or infected soft tissues [3]. An additional 10 cases were classified as “other” (e.g., intracranial abscess, meningitis, pancreatitis). Because multiple patients had more than one infection site, the total number of causes exceeded the number of patients. The identified pathogens included *Klebsiella pneumoniae*,* Escherichia coli*,* Pseudomonas aeruginosa*,* Acinetobacter baumannii complex*,* Candida albicans*,* Enterococcus faecalis*,* Staphylococcus aureus*,* Proteus mirabilis*,* Candida glabrata*,* Enterobacter cloacae complex*,* Staphylococcus epidermidis*,* Klebsiella oxytoca*, and *Serratia marcescens*. In some patients, different pathogens were isolated from one or multiple fluids or sources.

Over the study period, nutrition was predominantly enteral: ~50–57% of patients received enteral nutrition (EN), 23–27% received parenteral nutrition (PN), and 10–18% received a mixed EN and PN. In some cases, no nutritional treatment was provided (0–7%, depending on the time point). Detailed data are provided in the Supplementary Materials (Table S2). CRRT was applied in 30% of patients at baseline and in 13–17% thereafter. The therapy was performed exclusively in the continuous veno-venous hemodiafiltration (CVVHDF) modality with citrate predilution (18 mmol/l). Neither parameter showed any differences in proportions over time (χ² = 7.42, *p* = 0.829, and χ^2^ = 6.231, *p* = 0.518).

### Time-dependent changes in clinical and metabolic parameters modelled using GEE

#### Clinical scoring systems

Overall, patients exhibited progressive clinical improvement throughout the study period, as shown by model-based estimates. SOFA scores decreased significantly on Day 3 and Day 5 (*p* < 0.05), and APS score declined on Day 2 (*p* = 0.003) and remained stable (Figure S2A-B). The NUTRIC score, an indicator of nutritional risk, showed a similar trend, as a sustained decrease starting from Day 2 (Figure S2C), followed by stabilisation at subsequent time points was observed. RASS scores remained consistently low ( ~ − 4), confirming deep sedation across all time points. Detailed statistical results related to clinical scoring systems are provided in the Supplementary Materials (Table S3).

#### Inflammatory parameters

Markers of systemic inflammation modelled using GEE showed a delayed but marked decrease over time. CRP levels (Figure S3A) significantly declined on Day 5 and Day 7 (*p* < 0.01), and PCT (Figure S3B) followed a similar pattern. IL-6 levels (Figure S3C) decreased on Day 5 and Day 7 as well (both *p* < 0.05), with no significant changes observed earlier. As presented in Fig. 2D, WBC counts showed a modest reduction on Day 7 (*p* = 0.021), consistent with resolving inflammation. Detailed statistical results related to inflammatory markers are provided in the Supplementary Materials (Table S4).

#### Metabolic parameters

Baseline metabolic parameters were assessed on Day 1 and tracked throughout the observation period to evaluate trends over time using GEE models. Among metabolic markers, TC (Figure S4A) and LDL-C (Figure S4B) rose significantly on Day 5 and Day 7 (both *p* < 0.01) compared to baseline. Lactate dropped markedly (Figure S4C) from Day 2 onward (*p* < 0.01), while arterial blood pH (Figure S4D) increased significantly on Day 2 and stabilised thereafter. Other parameters, including HDL-C, glucose, triglycerides, and albumin, remained stable. Model-based estimates with corresponding SE are presented in Figure S4 and Table S5 (Supplementary Materials).

#### Nutritional parameters

Model-based estimates of nutritional parameters with corresponding SE are presented in Figure S5, with detailed data in Table S6 (Supplementary Materials). Nutritional intake showed dynamic changes. Energy (Figure S5A) and protein intake (assessed per kilogram of ABW, Figure S5E, and IBW, Figure S5F) increased significantly on Day 3 (*p* < 0.01), with protein remaining elevated on Day 5, after which their drop was observed. The protein-to-non-protein calorie ratio (Figure S5B) also peaked on Day 3 and Day 5. REE coverage (Figure S5D) followed a similar pattern, peaking on Day 3 before declining. In contrast, non-nutritional energy share (Figure S5C) significantly decreased starting from Day 3. Vitamin A and E intake increased temporarily but did not maintain statistical significance after multiple comparison adjustments. No significant changes were detected for vitamin D, K, C, or B-group vitamins.

### Multivariable modelling

#### Baseline models

In the first step, changes in REE and RQ over time were analysed using LMMs, which included time (categorical), age and sex as fixed effects. These covariates were selected a priori based on their established relevance to REE and clinical plausibility. The model also included a random intercept for patient identifier (ID) and assumed an AR (1) autocorrelation structure for repeated measures (Equation S1, Supplementary material). Baseline mixed-effect models for the mentioned parameters are presented in Table 2.

**Table 2:** Estimated fixed effects from the baseline linear mixed-effects model for resting energy expenditure (REE) and respiratory quotient (RQ)

Predictor	β + SE	
	REE	RQ
Intercept (β_0_)	2358.7 ± 29.35 ^***^	0.769 ± 0.052 ^***^
Day 2	36.60 ± 12.261	0.011 ± 0.001
Day 3	105.8 ± 22.22	0.008 ± 0.00011
Day 5	163.7 ± 27.523 ^*^	0.071 ± 0.008 ^*^
Day 7	-46.8 ± 9.66	0.059 ± 0.031 ^#^
Age	-9.718 ± 1.117 ^*^	(-6.2 ± 0.69)·10^− 4^
Sex (Male)	235.9 ± 37.00 ^#^	-0.025 ± 0.002
σ^2^_intercept_	971	0.012
σ^2^_residual_	553	0.018
R^2^_marginal_	0.228	0.081
R^2^_conditional_	0.704	0.411

The model includes time (categorical), age, and sex as fixed effects; a random intercept for patient identifier (ID) measured by σ^2^_intercept_; and an AR (1) autocorrelation structure to account for repeated measurements over time measured by σ^2^_residual_. The intercept represents the estimated value for the reference group (Day 1, female sex, age = 0, as defined by the model). Age is modelled as a continuous variable. Standard errors and p-values are based on restricted maximum likelihood estimation (REML)

*** for *p* < 0.001, ** for *p* < 0.01, * for *p* < 0.05, # for *p* < 0.1; R^2^_marginal_ – the variance explained by fixed effects, R^2^_conditional_ - the variance explained by both fixed and random effects. REE - resting energy expenditure; RQ - respiratory quotient

The baseline models explained 22.8% of the variance in REE and 8.1% in RQ via fixed effects alone (marginal R² equal to 0.228 and 0.081), and 70.4% and 41.1% when including both fixed and random effects (conditional R² equal to 0.704 and 0.411).

The baseline REE for the reference group (Day 1, Age = 0, and Sex = F) was estimated at 2358.7 kcal/day. Over time, changes in REE were observed, with a statistically significant increase detected on Day 5 (estimate = 163.682, *p* = 0.049) compared to the baseline, corresponding to an increase of approximately 164 kcal/day. No statistically significant changes were found on Day 2, 3, or 7. Age was significantly associated with REE, with an estimated decrease of -9.718 kcal/day per year (*p* = 0.026), indicating a gradual decline in REE with increasing age. Additionally, sex-related differences were observed, as male patients had a mean REE of approximately 236 kcal/day higher than females; however, this difference did not reach statistical significance (*p* = 0.097).

**Fig. 1:**
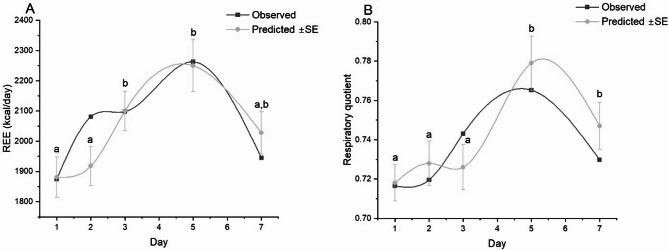
Changes in REE (A) and RQ (B) over the observation time points presented as medians (for observed values) and estimates (predicted) according to a mixed-effects model adjusted for covariates. Error bars represent standard errors (SE). According to pairwise comparisons, time points not sharing the same letter (a, b) differ significantly in the level of the variable (*p* < 0.05). Number of patients per time point: Day 1 (*n* = 30), Day 2 (*n* = 22), Day 3 (*n* = 22), Day 5 (*n* = 18), Day 7 (*n* = 13)

The baseline RQ for the reference group (Day 1, Age = 0, and Sex = F) was estimated at 0.769. Over time, RQ increased significantly on Day 5 (β = 0.071, *p* = 0.013) compared to baseline. A borderline increase was observed on Day 7 (estimate = 0.059, *p* = 0.065). At the same time, no statistically significant differences were found at Days 2 or 3. Age (*p* = 0.375) and sex (*p* = 0.311) were not significantly associated with RQ. Pairwise comparisons, adjusted for multiple testing using the Bonferroni correction, revealed that RQ values at Days 5 and 7 differed significantly from those at other time points at *p* < 0.05. Figure 1 illustrates the temporal trajectories of observed and model-predicted values for REE (A) and RQ (B) during the first week of sepsis.

All three metabolic parameters demonstrated a progressive rise from Day 1, peaking between Days 3 and 5, followed by a subsequent decline by Day 7. Model predictions closely approximated observed values, particularly on Days 1, 3, and 5, with larger variability noted on Day 7, likely due to smaller sample sizes and increased clinical heterogeneity at this time point. Statistically significant differences between time points, as determined by pairwise comparisons of estimated marginal means, are indicated by distinct letters (a, b). These differences were most pronounced during the peak of the hypermetabolic response (Days 3–5), underscoring the dynamic and non-linear nature of energy metabolism during early sepsis.

### Influence of clinical and biochemical variables on energy metabolism

A broad panel of clinical and biochemical variables was assessed using individual LMMs to identify specific contributors to REE and RQ variability. All models were adjusted for age and sex, and autocorrelation (AR (1)) structure to account for patient-level variability and repeated measures across time and each clinical or nutritional parameter was added one at a time as a fixed effect. No interaction terms were included. Variables tested included those with clinical plausibility or known relevance to metabolism in critical illness (e.g., temperature, lactate, CRRT, arterial pH, LDL-C, glucose, various nutritional intake metrics, and select vitamins). The list of all tested variables and key statistical parameters from the LMM analysis is summarised in Tables S7 and S8 (Supplementary Materials).

### Resting energy expenditure

**Fig. 2:**
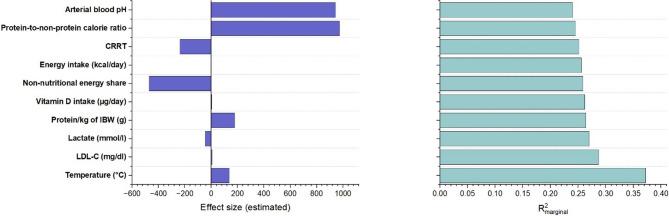
Dual-panel summary of predictors associated with the REE in ICU patients with sepsis. Left panel: Effect sizes (β estimates) from linear mixed-effects models (LMMs). Right panel: Corresponding marginal R² values indicating the proportion of variance explained by fixed effects. Statistically significant associations (*p* < 0.05) are shown in blue

The most influential predictors of REE assessed according to the significance level and R^2^_marginal_ (Fig. 2), which measures the explained portion of factor variability, were temperature, CRRT, lactate, arterial blood pH, a few nutrition-related factors (protein-to-non-protein calorie ratio, non-nutritional energy share, energy intake, protein intake per kilogram of IBW and vitamin D intake). Temperature emerged as the strongest positive predictor of REE (estimate = + 136.3 kcal/°C, *p* < 0.001; R² _marginal_ = 0.372). Even moderate increases in body temperature translated into substantial rises in REE. In contrast, patients undergoing CRRT exhibited significantly lower REE values (− 237.1 kcal/day, *p* = 0.036; R² = 0.251), indicating that patients receiving CRRT had REE values approximately 237 kcal/day lower. Among metabolic stress markers, only LDL-C, lactate, and arterial blood pH explained a significant part of REE variability (R²_marginal_ = 0.287, R²_marginal_ = 0.270, and 0.240, respectively). Lactate was inversely associated with REE (− 45.1 kcal/mmol/l, *p* = 0.023), while LDL-C and arterial blood pH showed positive association with REE, being strong for pH (+ 942.9 kcal/unit, *p* = 0.013). Nutritional factors turned out to be key contributors, particularly the protein-to-non-protein calorie ratio (R²_marginal_ = 0.245) and the non-nutritional energy share (R²_marginal_ = 0.259). The protein-to-non-protein calorie ratio was also strongly associated with increased REE (+ 972.1 kcal/unit, *p* = 0.006). In the same time, a higher non-nutritional energy share was linked with reduced REE (− 470.4 kcal/%, *p* = 0.021). In contrast, energy intake (+ 0.139 kcal/kcal, *p* = 0.024) and protein intake per kilogram of IBW (+ 178.4 kcal/g, *p* = 0.028) demonstrated the positive interaction with REE. Among the studied vitamins, only vitamin D intake was significantly associated with REE (β = 3.111, *p* = 0.008), suggesting that for each one µg/day increase in vitamin D level, REE increased by approximately 3.1 kcal/day.

High conditional R^2^ values (up to 0.75) underscore the substantial inter-individual variability in REE, suggesting that patient-specific factors beyond the measured clinical variables also contribute meaningfully to energy expenditure.

Based on the estimated marginal means of REE at baseline (Day 1: 2358.7 ± 29.35 kcal) and at its peak (Day 5: 2522.7 ± 27.52 kcal), the effect size (f = 0.756) was calculated. For a repeated measures design with one group, five time points, correlation among repeated measures = 0.5, and nonsphericity correction ε = 0.75, the statistical power exceeded 0.99, indicating that the study was sufficiently powered to detect changes in REE over time.

### Respiratory quotient

**Fig. 3:**
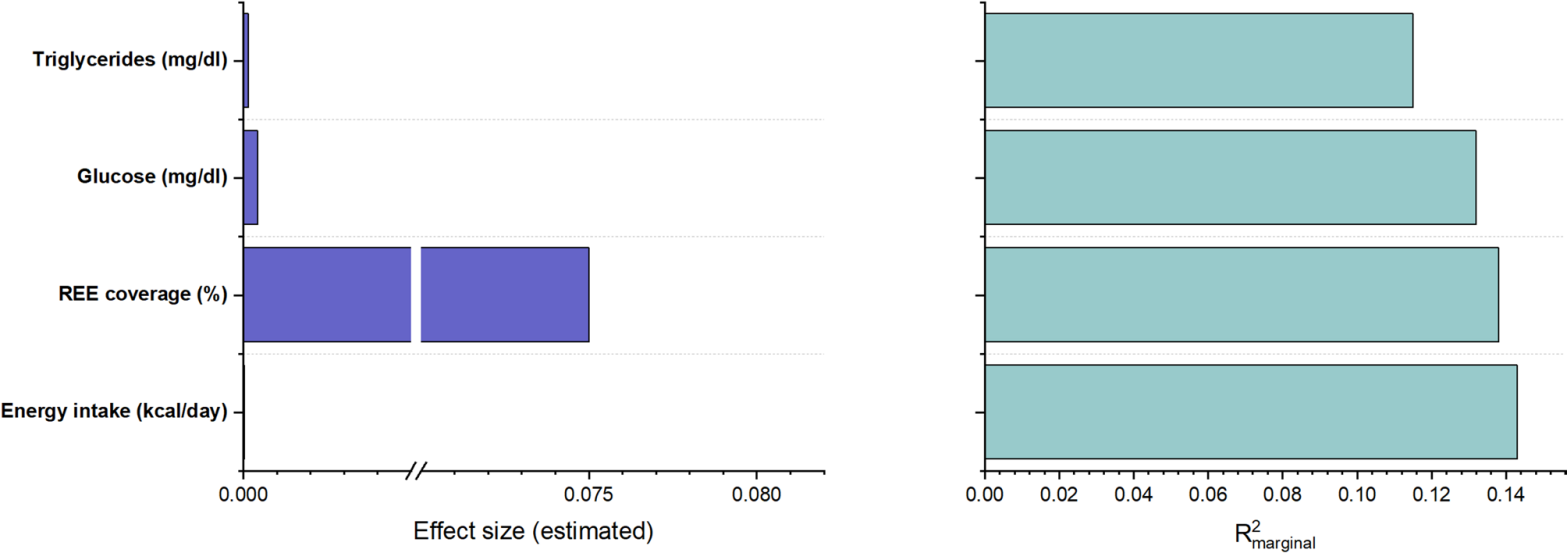
Dual-panel summary of predictors associated with the RQ in ICU patients with sepsis. Left panel: Effect sizes (β estimates) from linear mixed-effects models (LMMs). Right panel: Corresponding marginal R² values indicating the proportion of variance explained by fixed effects. Statistically significant associations (*p* < 0.05) are shown in blue

Although most effect sizes were modest, several variables were significantly associated with RQ (Fig. 3). Nutritional parameters emerged as primary contributors. Energy intake showed a significant positive association with RQ (β = 4.13 × 10⁻⁵, *p* = 0.016), as did REE coverage (β = 0.075, *p* = 0.028). Among metabolic markers, only blood glucose level (β = 4.3 × 10⁻⁴, *p* = 0.025) demonstrated significant associations, while triglycerides (β = 1.5 × 10⁻⁴, *p* = 0.057) showed a borderline effect.

Other clinical variables, including temperature, CRRT, inflammatory markers, and severity scores, were not significantly associated with RQ. Although some predictors (e.g., albumin, PCT, HDL-C) showed moderate R²_marginal_ values, they did not reach statistical significance.

Overall, energy intake, REE coverage, blood glucose levels, and marginally triglycerides were the primary determinants of RQ, while inflammatory markers, clinical scores, and protein intake had minimal association. A post-hoc power analysis for RQ was based on estimated marginal means on Day 1 (0.769 ± 0.052) and Day 5 (0.840 ± 0.008). The effect size (f = 0.306) was moderate, and the corresponding statistical power was estimated to exceed 0.85.

## Discussion

### Resting energy expenditure

The results of this study provide essential insights into the metabolic changes occurring in patients with sepsis over consecutive days of hospitalisation in the ICU. Significant variations in REE were observed, in line with the established metabolic response pattern in sepsis [3]. The initial increase in REE may indicate the presence of a hypermetabolic phase, consistent with previous findings describing an intense activation of catabolic processes in critically ill patients. Following the initial increase, the subsequent gradual decline in REE over time may suggest a transition toward a hypometabolic phase in some patients, possibly reflecting metabolic adaptation; however, this pattern was not consistent across the entire cohort.

The analysis of clinical factors revealed a significant association between temperature and REE, confirming the well-documented relationship between febrile states and increased metabolic demands [24, 25]. The positive correlation between temperature and REE observed in this study suggests that increased body temperature directly contributes to elevated energy expenditure in patients with sepsis. This suggests a potential role of fever control strategies in modulating metabolic stress and optimising energy balance in critically ill individuals.

A strong correlation between CRRT and REE was observed, with patients undergoing CRRT exhibiting significantly lower REE than those not receiving CRRT. On average, REE in CRRT patients was 237 kcal/day lower, suggesting that CRRT influences energy metabolism. Notably, this difference occurred despite the application of Jonckheer-Wasyluk correction formulas adjusting Weir’s equation for CO₂ loss during CRRT [23]. Several mechanisms may explain this phenomenon. As indicated in the study by Jonckheer et al., CRRT can lead to both the unintended delivery of calories (e.g., from citrate and glucose in dialysate fluids) and their loss, depending on the treatment settings [26, 27]. Moreover, CRRT may reduce systemic metabolic stress by clearing circulating cytokines, metabolic by-products, and pro-inflammatory mediators, affecting overall energy demand [28, 29]. Additionally, CRRT induces heat loss and immunologic activation, which may further alter actual REE [26], highlighting the complex interplay between extracorporeal support and metabolic regulation.

Lactate levels decreased markedly from Day 2 onward, while arterial blood pH increased significantly and remained stable thereafter. These changes suggest early improvement in acid–base balance and systemic metabolic recovery. Among metabolic stress markers, lactate and arterial blood pH were the only variables showing a significant association with REE, suggesting a potential link between acid–base status and energy metabolism in sepsis. Elevated lactate and acidosis in sepsis may result from impaired tissue perfusion, mitochondrial dysfunction, or a shift toward anaerobic metabolism. Mitochondrial impairment – referred to as cytopathic hypoxia – is characterised by reduced cellular oxygen utilization despite adequate oxygen delivery, leading to impaired adenosine triphosphate (ATP) production [3, 30, 31]. As tissue perfusion improves and acid–base status normalizes, a shift toward more efficient substrate utilization and increased metabolic activity likely occurs. This transition aligns with the progression from the hypometabolic “ebb” phase to the hypermetabolic “flow” phase commonly described in the pathophysiology of sepsis.

Notably, LDL-C levels were positively correlated with REE, suggesting a potential link between lipid metabolism and energy expenditure in sepsis. However, the relatively low explanatory power observed in the model suggests that LDL-C is unlikely to be a key driver of REE and may instead reflect broader alterations in lipid homeostasis during critical illness. Previous studies have demonstrated that lower LDL-C levels on ICU admission are associated with increased mortality in critically ill patients, including those with sepsis [32].

Energy and protein intake varied throughout the observation period, likely reflecting clinical instability and evolving nutritional strategies. The highest energy intake was observed on Day 3, followed by a gradual decline in later time points. Similarly, protein intake per kilogram of body weight peaked on Day 3 and Day 5 before decreasing. These fluctuations likely reflect the challenges of optimising nutritional support in critically ill patients, particularly in dynamic metabolic changes and varying clinical conditions. Our findings suggest a significant correlation between energy intake, protein intake, and the protein-to-non-protein calorie ratio with REE. Specifically, higher protein intake was correlated with an increase in REE, while a higher non-nutritional energy share was associated with a decrease in REE. One potential explanation for these trends is the gradual increase in the administration of nutritional treatment products alongside a decreasing reliance on non-nutritional energy sources, such as glucose solutions and propofol in lipid emulsion form, throughout hospitalisations. This shift may indicate a more structured nutritional management approach as patients stabilise. Current guidelines emphasise early and progressive EN in patients with sepsis after hemodynamic stabilisations, with PN as a supplemental option if EN is contraindicated. Given the risks associated with underfeeding and overfeeding, a pragmatic approach recommends initiating EN within the first three to four days after ICU admission with a fraction (20–50%) of full nutrition support to “open” the enteral route. The amounts of feeds should then be progressively increased according to gastrointestinal tolerance to achieve optimal nutritional support as patients improve. If this is not feasible for prolonged periods, PN should be prescribed to supplement energy intake. Additionally, in septic shock, impaired splanchnic perfusion may necessitate delaying EN until successful resuscitation, reinforcing the importance of individualised nutrition strategies in critically ill patients [16].

Among the analysed vitamins, vitamin D intake emerged as the only variable significantly associated with REE, suggesting a potential role in energy metabolism regulation in critically ill patients. This aligns with ESPEN guidelines, emphasising that critically ill patients are at high risk of vitamin D deficiency. Such deficiency has been linked to prolonged ICU stays, higher incidence of sepsis-related complications, and increased mortality. Given vitamin D’s role in metabolic regulation through its nuclear receptor and its involvement in immune and inflammatory responses, its intake may influence energy metabolism in critically ill patients [16, 33, 34]. However, despite the observed association, there remains uncertainty regarding the optimal dosing and timing of vitamin D administration in this population, underlining the need for further studies to clarify its therapeutic potential in sepsis management.

### Respiratory quotient

The RQ, defined as the ratio of V̇CO₂ to V̇O₂, reflects the predominant type of metabolic substrate utilisation. An RQ value of 1.0 indicates predominant glucose oxidation, whereas values close to 0.7 suggest fat oxidation; a further decrease below 0.7 may indicate the utilisation of ketone bodies. Under physiological conditions, RQ typically ranges from 0.67 to 1.3, with a standard mixed oral diet generating an RQ value of approximately 0.8 [35, 36, 37, 38].

In the present study, a significant increase in RQ was observed over time, potentially reflecting a shift toward greater carbohydrate oxidation in the later stages of sepsis. This trend may result from changes in substrate availability and progressing metabolic adaptation. Furthermore, the positive correlations between RQ and energy intake and RQ and the degree of REE coverage suggest that the administered nutritional substrates were actively utilised in oxidative metabolism, potentially contributing to increased carbohydrate oxidation. Importantly, elevated RQ values approaching or exceeding 1.0 may also indicate excessive caloric intake, particularly from carbohydrate-rich sources, which can enhance lipogenesis and increase CO₂ production [39–41]. In mechanically ventilated patients – especially those with sepsis-associated acute respiratory distress syndrome (ARDS) – this may impose additional burden on respiratory function and complicate weaning from ventilatory support [42].

Moreover, non-nutritional factors may influence RQ values, including CO₂ retention, disturbances in acid–base balance, and mechanical ventilator settings. For example, inadequate alveolar ventilation may lead to CO₂ accumulation, artificially elevating RQ values. Ventilator parameters such as tidal volume, respiratory rate, and dead space ventilation also directly affect CO₂ elimination and should be carefully considered when interpreting RQ results [39, 43–45].

Despite the fact that overfeeding is not the only factor influencing RQ, its potential role in metabolic monitoring cannot be overlooked. With the increasing availability of metabolic modules, continuous bedside monitoring of RQ has become feasible, positioning it as a potential marker for overfeeding in critically ill patients, with this topic representing an area for further investigation.

### Limitations and future directions

Several limitations should be acknowledged. This study was conducted in a single centre with a relatively small sample size, which may limit the generalizability of the findings and reduce the statistical power for detecting certain associations. The observational nature of this study also restricts causal interpretations regarding metabolic parameters and clinical outcomes. While IC is the gold standard for energy expenditure measurement, its application in critically ill patients was affected by technical challenges, such as ventilator settings and oxygen supplementation. These factors led to the exclusion of some patients with sepsis or the premature termination of their participation, which may have influenced the dataset. Consequently, patients at the most severe end of the clinical spectrum – e.g., those requiring high FiO₂ or experiencing non-steady-state ventilatory conditions – were often ineligible for reliable IC and are likely underrepresented; this could bias estimates away from the extremes seen in the earliest, most unstable phase of sepsis. Moreover, the intervals from hospital admission to study inclusion and from ICU admission to inclusion varied across patients; although reported in the Results, this heterogeneity may confound the interpretation of longitudinal trajectories anchored at sepsis onset. ICU admission time alone may be a misleading metabolic reference point, because pre-ICU hospitalization – regardless of ward (medical, surgical or trauma) – can independently perturb metabolism. Additionally, metabolic measurements were performed at specific time points, limiting the ability to capture continuous changes in energy metabolism. More frequent metabolic assessments in future studies could provide a better understanding of these dynamics. The lack of a significant correlation between REE and glucose levels may be explained by the influence of therapeutic interventions, including intravenous glucose administration and insulin therapy, which regulate glucose metabolism and might obscure direct associations. Other pharmacological treatments, such as vasoactive drugs and sedatives, may have also impacted metabolic parameters but were not explicitly controlled for in this analysis. Another limitation concerns route-specific bioavailability: patients receiving EN, PN, or mixed feeding were analysed within the same framework, yet EN may be partially unabsorbed due to gastrointestinal losses in critical illness, whereas PN is typically fully bioavailable [46].

Further research is needed to clarify the regulatory mechanisms underlying metabolic changes in sepsis and the impact of nutritional therapy. Future studies should focus on identifying factors determining individual variability in REE and assessing the long-term metabolic consequences in sepsis survivors. Animal models could provide valuable insights under controlled conditions, helping to explore long-term metabolic adaptations. Additionally, investigating the optimal timing and intensity of nutritional interventions could contribute to better metabolic outcomes in patients with sepsis.

## Conclusions

This study highlights the dynamic changes in energy metabolism during sepsis. The findings underscore the limitations of predictive equations in estimating energy demands and emphasise the need for individualised metabolic assessments. Significant correlations between REE and temperature, CRRT, arterial blood pH, lactate levels, and nutritional treatment strategies provide valuable insights into regulating metabolism in sepsis. The obtained results suggest the necessity of optimising metabolic and nutritional support and indicate directions for future research on long-term metabolic consequences in patients with sepsis. Future studies should aim to establish the causality of these correlations and confirm these findings in a larger patient cohort. Moreover, the potential application of continuous bedside monitoring of RQ as a marker for overfeeding warrants further investigation, as it could offer a valuable tool for optimising nutritional strategies and improving patient outcomes in critically ill populations.

## Supplementary Information

Below is the link to the electronic supplementary material.

Supplementary Material 1

## Data Availability

Detailed results of the statistical analyses are provided in the Supplementary Materials. The raw datasets generated and analysed during the current study are not publicly available due to concerns regarding patient privacy but are available from the corresponding author upon reasonable request.

## Abbreviations

ABW: Actual body weight
ACF: Autocorrelation function
AIC: Akaike information criterion
APS: Acute Physiology Score
ARDS: Acute respiratory distress syndrome
AR(1): Autoregressive model of order 1
ATP: Adenosine triphosphate
BAL: Bronchoalveolar lavage
BIC: Bayesian information criterion
BMI: Body mass index
CO₂: Carbon dioxide
CRP: C-reactive protein
CRRT: Continuous renal replacement therapy
CVVHDF: Continuous veno-venous hemodiafiltration
EN: Enteral nutrition
EMM(s): Estimated marginal means
FiO₂: Fraction of inspired oxygen
GEE: Generalised estimating equations
Hb: Hemoglobin
HCT: Hematocrit
HDL-C: High-density lipoprotein cholesterol
IBW: Ideal body weight
IC: Indirect calorimetry
ICU: Intensive care unit
ID: Identifier
IL-6: Interleukin-6
IQR: Interquartile range
K⁺: Potassium
LDL-C: Low-density lipoprotein cholesterol
LMM(s): Linear mixed-effects model(s)
MAR: Missing at random
MCAR: Missing completely at random
Na⁺: Sodium
NUTRIC: Nutrition Risk in Critically Ill
N₂: Nitrogen
O₂: Oxygen
pCO₂: Partial pressure of carbon dioxide
pO₂: Partial pressure of oxygen
PCT: Procalcitonin
PEEP: Positive end-expiratory pressure
PLT: Platelet count
PN: Parenteral nutrition
RASS: Richmond Agitation–Sedation Scale
REE: Resting energy expenditure
REML: Restricted maximum likelihood estimation
RQ: Respiratory quotient
R²_marginal_: Variance explained by fixed effects
R²_conditional_: Variance explained by both fixed and random effects
SE: Standard error
SmPC: Summary of Product Characteristics
SOFA: Sequential Organ Failure Assessment
STROBE: Strengthening the Reporting of Observational Studies in Epidemiology
TC: Total cholesterol
VIF: Variance inflation factor
V̇CO₂: Carbon dioxide production
V̇O₂: Oxygen consumption
WBC: White blood cell count
