# Supplementary material for: Time-course changes in energy expenditure in sepsis: a prospective observational study
Source: Ann Intensive Care. 2025 Oct 14;15:158. doi: 10.1186/s13613-025-01592-3 (PMC12521711; doi:10.1186/s13613-025-01592-3)
Supplement: Supplementary file 1 — Supplementary Material 1 [file 13613_2025_1592_MOESM1_ESM.docx]

**Time-course changes in energy expenditure in sepsis: a prospective observational study**

Supplementary materials

**Equation S1.**

$$X_{ij}=\beta_{0}+\beta_{1}{Time}_{ij}+\beta_{2}{Age}_{i}+\beta_{3}{Sex}_{i}+\sigma_{intercept}^{2}+\sigma_{residual}^{2}$$

Where:

X_ij_ – estimated value of variable for patient i and time j,

β_0_ – fixed intercept,

β_1-3_– fixed effects of time, age and sex,

σ^2^_intercept_ – random intercept for patient i,

σ^2^_residual_ – residuals modelled with AR(1) correlation structure

**Table S1.** Definitions and calculation methods of variables used in the analysis.

| Variable | Definition | Measurement/Calculation |
| --- | --- | --- |
| Actual body weight (ABW) | The patient’s body weight measured daily using a bed scale | Measured once daily using the integrated scale in the ICU bed |
| Ideal body weight (IBW) | The weight corresponding to a BMI of 25 kg/m² | Calculated based on the patient’s height (H) using the formula:  $IBW [kg]=25 \times H^{2}$  where H is expressed in meters. |
| Body mass index (BMI) | Body mass index is a value derived from the mass and height | Calculated based on the patient’s height (H) and actual body weight (ABW) using the formula: $BMI \left[ \frac{kg}{m^{2}} \right]= \frac{ABW}{H^{2}}$  where H is expressed in meters and ABW in kilograms. |
| Energy intake | Total amount of energy received from all sources per day | Sum of energy delivered via nutritional sources (enteral and parenteral nutrition) and non-nutritional sources (e.g., medications), calculated in kilocalories per day based on product composition from drug labels, SmPCs, manufacturer websites, or direct contact with manufacturers when necessary |
| Protein-to-non-protein calorie ratio | The proportion of energy derived from protein relative to energy derived from non-protein sources | Calculated by dividing the energy from protein and amino acids by the combined energy from carbohydrates and fat, considering all nutritional and non-nutritional sources |
| Non-nutritional energy share | The proportion of total energy intake derived from non-nutritional sources | Calculated by dividing the energy from non-nutritional sources (e.g., medications) by the daily energy intake |
| REE coverage | The proportion of REE covered by energy intake | Calculated by dividing the daily energy intake by the REE measured using IC |
| Protein intake per kilogram of ABW and IBW | \| The amount of protein or amino acids administered per day, normalized to the patient's actual or ideal body weight \| \| --- \| | Calculated by dividing total daily protein or amino acid intake by ABW or IBW |
| Intake of vitamins | The amount of individual vitamins provided via enteral and parenteral nutrition or administered as other medications | Calculated as the sum of each vitamin from enteral nutrition, parenteral nutrition, and other medications based on product composition (e.g., SmPC or manufacturer data), expressed as a mass unit per day |

Abbreviations: ABW – actual body weight; IBW – ideal body weight; BMI – body mass index; H – height; IC – indirect calorimetry; REE – resting energy expenditure, SmPC – Summary of Product Characteristics.

**Figure S1.** Patient flow across study time points. Abbreviations: FiO₂ — fraction of inspired oxygen; IMV — invasive mechanical ventilation.


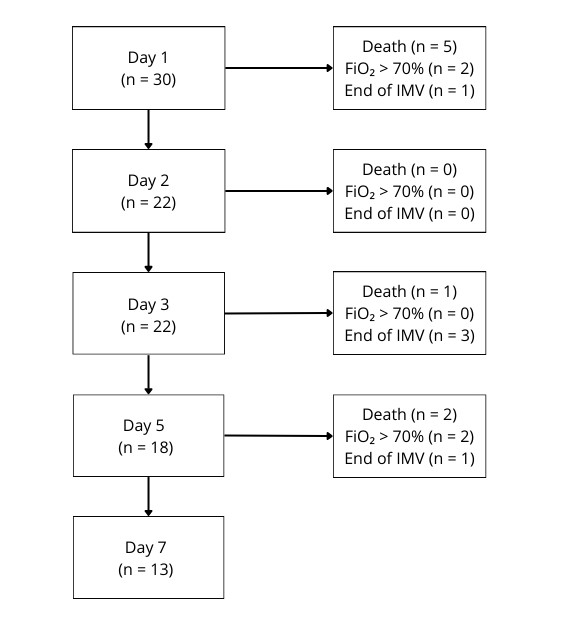


**Table S2.** Medians with interquartile ranges (IQR) for energy and macronutrient intake across five measurement time points (Day 1 – Day 7). Measurements are presented for each time point, along with the corresponding sample sizes (N).

| **Variable** | **Measurement** | **N** | **Median** | **IQR** |
| --- | --- | --- | --- | --- |
| Energy intake (kcal/day) | Day 1 | 30 | 1249 | 922–1585 |
|  | Day 2 | 22 | 1812 | 1474–2074 |
|  | Day 3 | 22 | 1924 | 1723–2122 |
|  | Day 5 | 18 | 1776 | 1553–1964 |
|  | Day 7 | 13 | 1575 | 1153–1762 |
| Protein intake (g/day) | Day 1 | 30 | 90.1 | 80.1–132.3 |
|  | Day 2 | 22 | 122.5 | 92.0–142.3 |
|  | Day 3 | 22 | 134.8 | 88.2–153.7 |
|  | Day 5 | 18 | 130.0 | 78.4–151.1 |
|  | Day 7 | 13 | 88.8 | 64.3–134.9 |
| Carbohydrate intake (g/day) | Day 1 | 30 | 114.3 | 80.8–133.4 |
|  | Day 2 | 22 | 146.0 | 108.8–163.2 |
|  | Day 3 | 22 | 156.4 | 112.9–174.7 |
|  | Day 5 | 18 | 125.0 | 98.1–148.4 |
|  | Day 7 | 13 | 122.5 | 82.5–153.1 |
| Sugars intake (g/day) | Day 1 | 30 | 80.0 | 61.2–112.0 |
|  | Day 2 | 22 | 97.5 | 78.0–115.1 |
|  | Day 3 | 22 | 102.5 | 87.1–126.5 |
|  | Day 5 | 18 | 60.2 | 49.1–81.9 |
|  | Day 7 | 13 | 59.0 | 41.6–72.0 |
| Lipid intake (g/day) | Day 1 | 30 | 53.5 | 39.7–75.1 |
|  | Day 2 | 22 | 65.9 | 56.1–87.7 |
|  | Day 3 | 22 | 73.2 | 52.6–95.0 |
|  | Day 5 | 18 | 67.5 | 39.2–73.3 |
|  | Day 7 | 13 | 47.4 | 29.7–55.1 |
| Protein energy intake (kcal/day) | Day 1 | 30 | 334.6 | 292.0–378.1 |
|  | Day 2 | 22 | 446.4 | 393.1–502.3 |
|  | Day 3 | 22 | 552.3 | 488.2–593.7 |
|  | Day 5 | 18 | 480.0 | 428.8–503.2 |
|  | Day 7 | 13 | 360.6 | 294.4–384.9 |
| Non-protein energy intake (kcal/day) | Day 1 | 30 | 970 | 732–1198 |
|  | Day 2 | 22 | 1245 | 988–1367 |
|  | Day 3 | 22 | 1417 | 1095–1598 |
|  | Day 5 | 18 | 1215 | 990–1438 |
|  | Day 7 | 13 | 1038 | 891–1196 |
| Nutritional energy intake (kcal/day) | Day 1 | 30 | 1000 | 806–1239 |
|  | Day 2 | 22 | 1403 | 1225–1658 |
|  | Day 3 | 22 | 1640 | 1452–1812 |
|  | Day 5 | 18 | 1490 | 1384–1556 |
|  | Day 7 | 13 | 1099 | 990–1190 |
| Non-nutritional energy intake  (kcal/day) | Day 1 | 30 | 380.4 | 306.8–422.0 |
|  | Day 2 | 22 | 340.1 | 297.7–396.8 |
|  | Day 3 | 22 | 280.2 | 209.3–326.5 |
|  | Day 5 | 18 | 210.3 | 182.9–276.6 |
|  | Day 7 | 13 | 179.7 | 125.1–245.1 |

**Table S3.** Median (with interquartile ranges) levels of different scales and their estimated values are calculated using the Generalized Estimating Equations (GEE) model with statistics. Statistical significance is provided for comparisons with baseline (Day 1). Differences among time points are determined with pairwise comparison using Bonferroni adjustment.

| **Variable** | **Measurement** | **N** | **Median** | **IQR** | **Estimated (SE)** | **p-value vs. baseline (Day1)** |
| --- | --- | --- | --- | --- | --- | --- |
| SOFA score | Day 1 | 30 | 8.0 | 6.0–10.8 | 8.33^a^ (0.541) | baseline |
|  | Day 2 | 22 | 7.5 | 5.2–8.8 | 7.27^a^ (0.587) | 0.185 |
|  | Day 3 | 22 | 6.0 | 4.2–7.8 | 6.46^a,b^ (0.655) | 0.027 |
|  | Day 5 | 18 | 6.0 | 3.2–8.8 | 6.44^a,b^ (0.727) | 0.078 |
|  | Day 7 | 13 | 4.0 | 3.0–7.0 | 5.46^b^ (0.727) | 0.007 |
| APS score | Day 1 | 30 | 7.0 | 4.0–12.0 | 8.30^a^ (0.879) | baseline |
|  | Day 2 | 22 | 5.0 | 4.0–6.0 | 5.18^b^ (0.581) | 0.003 |
|  | Day 3 | 22 | 5.5 | 3.2–8.0 | 5.91^b^ (0.677) | 0.031 |
|  | Day 5 | 18 | 5.0 | 5.0–7.8 | 5.44^b^ (0.869) | 0.033 |
|  | Day 7 | 13 | 4.0 | 4.0–6.0 | 5.00^b^ (0.697) | 0.003 |
| RASS score | Day 1 | 30 | - 4.0 | -5.0–-4.0 | - 4.03 (0.233) | baseline |
|  | Day 2 | 22 | - 4.0 | -5.0–-4.0 | - 4.00 (0.265) | 0.925 |
|  | Day 3 | 22 | - 4.0 | -5.0–-4.0 | - 3.86 (0.282) | 0.643 |
|  | Day 5 | 18 | - 3.5 | -5.0–-1.8 | - 3.39 (0.272) | 0.205 |
|  | Day 7 | 13 | - 3.5 | -5.0–-3.0 | - 3.69 (0.303) | 0.539 |
| NUTRIC score | Day 1 | 30 | 4.5 | 3.2–6.0 | 4.61^a^ (0.371) | baseline |
|  | Day 2 | 22 | 4.0 | 3.0–4.8 | 3.64^b^ (0.337) | 0.044 |
|  | Day 3 | 22 | 4.0 | 2.2–4.0 | 3.67^b^ (0.373) | 0.045 |
|  | Day 5 | 18 | 4.0 | 3.0–5.0 | 3.78^b^ (0.373) | 0.068 |
|  | Day 7 | 13 | 4.0 | 3.0–5.0 | 3.64^b^ (0.432) | 0.071 |

IQR – interquartile range; a and b indices show differences between time points according to pairwise comparison.


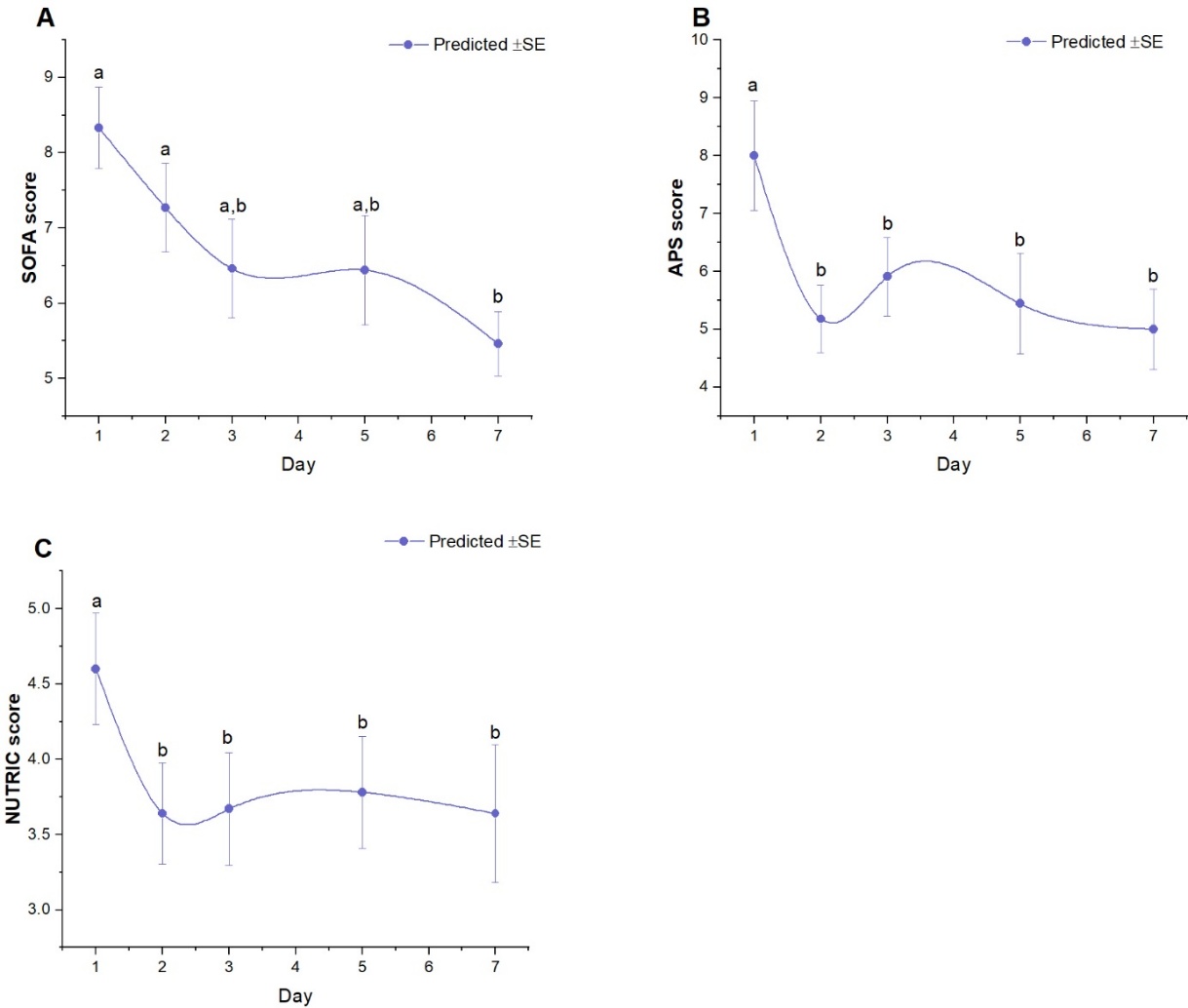


**Figure S2.** Model-based estimates of SOFA (A), APS (B), and NUTRIC (C) scores with SE. According to pairwise comparisons, time points not sharing the same letter (a, b) differ significantly in the level of the variable (p < 0.05). Number of patients per time point: Day 1 (n = 30), Day 2 (n = 22), Day 3 (n = 22), Day 5 (n = 18), Day 7 (n = 13).

**Table S4.** Inflammatory markers (CRP, PCT, Il-6 and WBC) medians (with IQR) and their estimated values (with SE) were calculated according to the Generalised Estimating Equations (GEE) model with statistics. Statistical significance is provided for comparisons with baseline (Day 1). Differences among time points are determined with pairwise comparison using Bonferroni adjustment.

| **Variable** | **Measurement** | **N** | **Median** | **IQR** | **Estimated (SE)** | **p-value vs. baseline (Day1)** |
| --- | --- | --- | --- | --- | --- | --- |
| CRP (mg/l) | Day 1 | 27 | 248.43 | 182.0–318.1 | 242.2^a^ (22.5) | baseline |
|  | Day 2 | 22 | 257.17 | 183.1–325.3 | 242.3^a^ (23.5) | 0.997 |
|  | Day 3 | 22 | 189.40 | 148.2–253.7 | 189.0^a,b^ (20.8) | 0.083 |
|  | Day 5 | 18 | 125.18 | 68.8–173.2 | 124.7^b^ (20.9) | < 0.001 |
|  | Day 7 | 13 | 120.30 | 74.4–184.9 | 123.2^b^ (27.1) | 0.003 |
| PCT (ng/ml) | Day 1 | 30 | 12.61 | 6.3–20.0 | 16.7^a^ (3.81) | baseline |
|  | Day 2 | 22 | 11.88 | 4.8–18.8 | 14.6^a^ (3.99) | 0.204 |
|  | Day 3 | 22 | 8.16 | 2.2–14.3 | 10.7^a^ (2.24) | 0.078 |
|  | Day 5 | 18 | 2.89 | 0.8–5.1 | 4.4^b^ (1.16) | 0.031 |
|  | Day 7 | 13 | 1.13 | 0.5–1.4 | 1.5^b^ (0.49) | 0.020 |
| IL-6 (pg/ml) | Day 1 | 27 | 201.25 | 147.0–315.6 | 282.7^a^ (53.3) | baseline |
|  | Day 2 | 19 | 178.60 | 41.6–132.0 | 171.4^a^ (38.8) | 0.160 |
|  | Day 3 | 15 | 154.20 | 108.0–185.8 | 154.7^a,b^ (34.3) | 0.161 |
|  | Day 5 | 14 | 90.46 | 27.1–156.5 | 90.5^b^ (37.3) | 0.031 |
|  | Day 7 | 7 | 90.90 | 29.1–141.6 | 90.7^b^ (39.4) | 0.037 |
| WBC (1000/µl) | Day 1 | 30 | 14.33 | 9.2–22.1 | 14.3^a^ (2.29) | baseline |
|  | Day 2 | 22 | 11.60 | 6.7–19.6 | 12.9^a^ (2.25) | 0.286 |
|  | Day 3 | 22 | 10.61 | 7.8–20.7 | 12.1^a^ (2.21) | 0.278 |
|  | Day 5 | 18 | 13.07 | 10.3–17.8 | 13.5^a^ (1.47) | 0.156 |
|  | Day 7 | 13 | 9.51 | 7.9–15.9 | 9.2^b^ (1.39) | 0.021 |

IQR – interquartile range; a and b indices show differences between time points according to pairwise comparison.

**
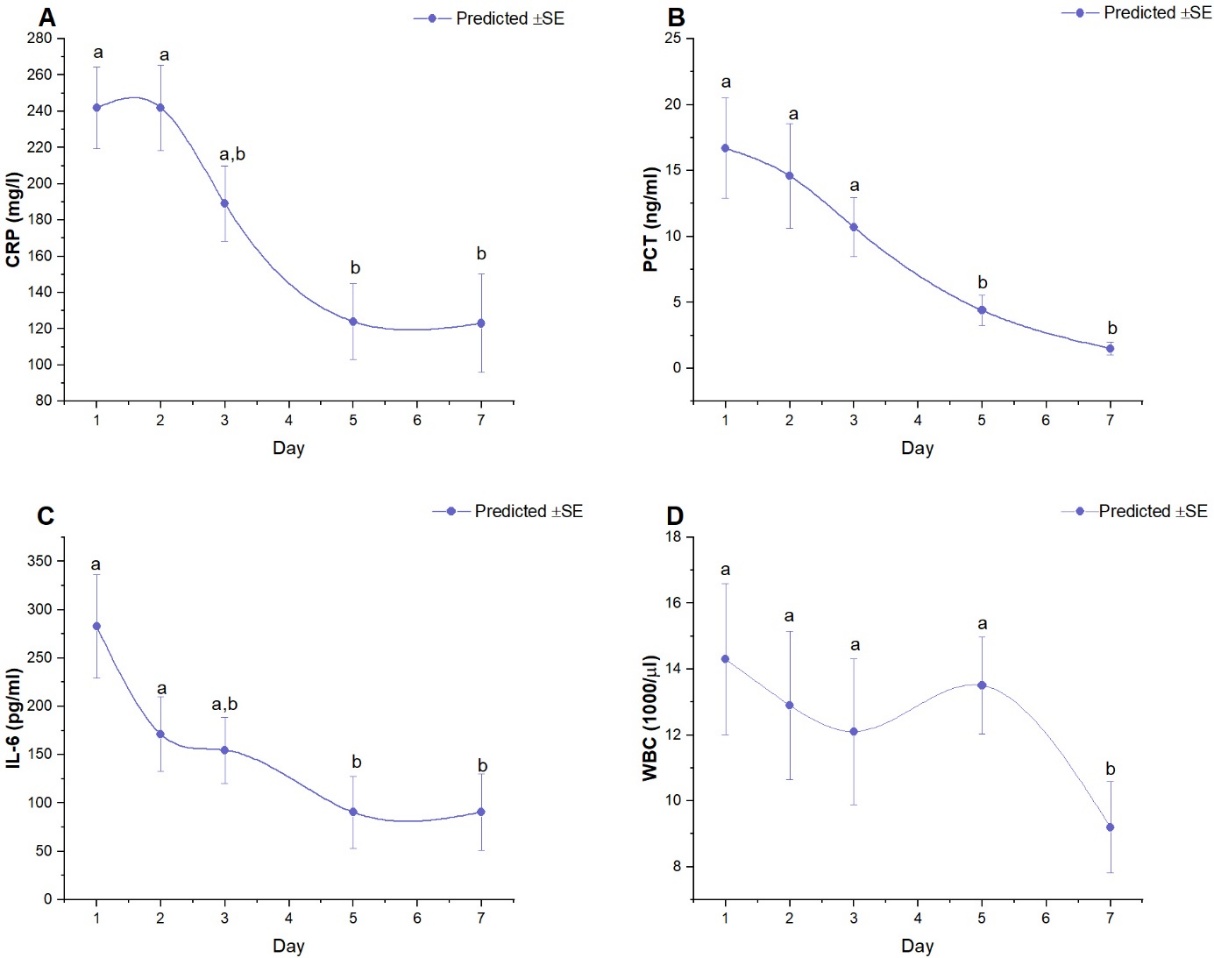
**

**Figure S3.** Model-based estimates of CRP (A), PCT (B), IL-6 (C), and WBC (D) levels with SE. According to pairwise comparisons, time points not sharing the same letter (a, b) differ significantly in the level of the variable (p < 0.05). Number of patients per time point: Day 1 (n = 30), Day 2 (n = 22), Day 3 (n = 22), Day 5 (n = 18), Day 7 (n = 13).

**Table S5.** Median values (with IQR) and model-based estimates (with standard error) of metabolic parameters over time calculated using the Generalised Estimating Equations (GEE) model. Statistical significance is provided for comparisons with baseline (Day 1). Differences among time points are determined with pairwise comparison using the Bonferroni adjustment.

| **Variable** | **Measurement** | **N** | **Median** | **IQR** | **Estimated (SE)** | **p-value vs. baseline (Day 1)** |
| --- | --- | --- | --- | --- | --- | --- |
| Albumin (g/dl) | Day 1 | 28 | 2.92 | 2.83–3.11 | 2.87 (0.06) | baseline |
|  | Day 2 | 21 | 2.88 | 2.82–3.04 | 2.93 (0.05) | 0.510 |
|  | Day 3 | 22 | 3.03 | 2.83–3.14 | 2.96 (0.06) | 0.300 |
|  | Day 5 | 18 | 3.03 | 2.74–3.25 | 2.98 (0.08) | 0.261 |
|  | Day 7 | 13 | 3.02 | 2.77–3.24 | 3.00 (0.07) | 0.173 |
| TC  (mg/dl) | Day 1 | 28 | 94.0 | 78.5–112.0 | 94.9^a^ (5.74) | baseline |
|  | Day 2 | 22 | 100.5 | 77.2–118.0 | 101.9^a^ (7.26) | 0.449 |
|  | Day 3 | 21 | 111.0 | 101.0–125.0 | 108.4^a^ (8.12) | 0.173 |
|  | Day 5 | 15 | 118.0 | 110.0–125.5 | 120.2^b^ (7.84) | 0.009 |
|  | Day 7 | 12 | 115.5 | 108.8–132.2 | 119.3^b^ (6.07) | 0.003 |
| LDL-C  (mg/dl) | Day 1 | 28 | 41.5 | 34.8–56.2 | 45.1^a^ (4.93) | baseline |
|  | Day 2 | 22 | 41.5 | 28.8–57.5 | 48.3^a^ (5.48) | 0.667 |
|  | Day 3 | 20 | 59 | 43.8–71.0 | 60.6^a,b^ (5.42) | 0.034 |
|  | Day 5 | 16 | 75.5 | 57.0–85.5 | 72.8^b^ (5.80) | < 0.001 |
|  | Day 7 | 12 | 74.5 | 67.0–85.5 | 80.0^b^ (6.12) | < 0.001 |
| HDL-C  (mg/dl) | Day 1 | 28 | 13.5 | 7.8–16.8 | 15.6 (1.89) | baseline |
|  | Day 2 | 22 | 12 | 7.8–17.8 | 13.7 (1.71) | 0.461 |
|  | Day 3 | 21 | 11 | 8.0–16.0 | 13.6 (1.87) | 0.461 |
|  | Day 5 | 15 | 16 | 9.0–20.5 | 16.4 (2.26) | 0.783 |
|  | Day 7 | 12 | 15 | 13.0–20.2 | 17.2 (2.34) | 0.580 |
| Triglyceride (mg/dl) | Day 1 | 29 | 171 | 131.0–208.0 | 198.1 (23.1) | baseline |
|  | Day 2 | 22 | 234.5 | 180.0–294.5 | 256.8 (30.2) | 0.191 |
|  | Day 3 | 21 | 226 | 205.0–308.0 | 245.0 (28.8) | 0.188 |
|  | Day 5 | 16 | 218 | 184.0–309.0 | 227.3 (29.9) | 0.582 |
|  | Day 7 | 12 | 175 | 107.0–313.8 | 180.7 (29.5) | 0.817 |
| Lactate (mmol/l) | Day 1 | 30 | 1.20 | 0.8–1.6 | 1.49^a^ (0.64) | baseline |
|  | Day 2 | 22 | 1.15 | 0.7–1.5 | 1.29^a,b^ (0.31) | 0.035 |
|  | Day 3 | 22 | 0.91 | 0.6–1.4 | 1.05^a,b^ (0.32) | 0.022 |
|  | Day 5 | 18 | 0.95 | 0.7–1.4 | 1.04^a,b^ (0.22) | 0.010 |
|  | Day 7 | 13 | 0.84 | 0.7–1.1 | 0.95^b^ (0.14) | 0.002 |
| Glucose (mg/dl) | Day 1 | 30 | 137 | 121.8–164.0 | 142.1 (8.08) | baseline |
|  | Day 2 | 22 | 132.5 | 119.0–162.5 | 140.9 (8.47) | 0.921 |
|  | Day 3 | 22 | 141 | 116.5–173.0 | 147.9 (11.0) | 0.672 |
|  | Day 5 | 18 | 136 | 110.5–183.2 | 143.0 (12.8) | 0.498 |
|  | Day 7 | 13 | 122 | 111.0–186.0 | 133.1 (19.7) | 0.633 |
| Arterial blood pH | Day 1 | 30 | 7.39 | 7.28–7.44 | 7.37^a^ (0.02) | baseline |
|  | Day 2 | 22 | 7.43 | 7.39–7.48 | 7.44^b^ (0.01) | 0.001 |
|  | Day 3 | 22 | 7.45 | 7.40–7.49 | 7.43^b^ (0.01) | 0.004 |
|  | Day 5 | 18 | 7.46 | 7.41–7.50 | 7.43^b^ (0.02) | 0.015 |
|  | Day 7 | 13 | 7.44 | 7.39–7.48 | 7.45^b^ (0.01) | < 0.001 |

IQR – interquartile range; a and b indices show differences between time points according to pairwise comparison.


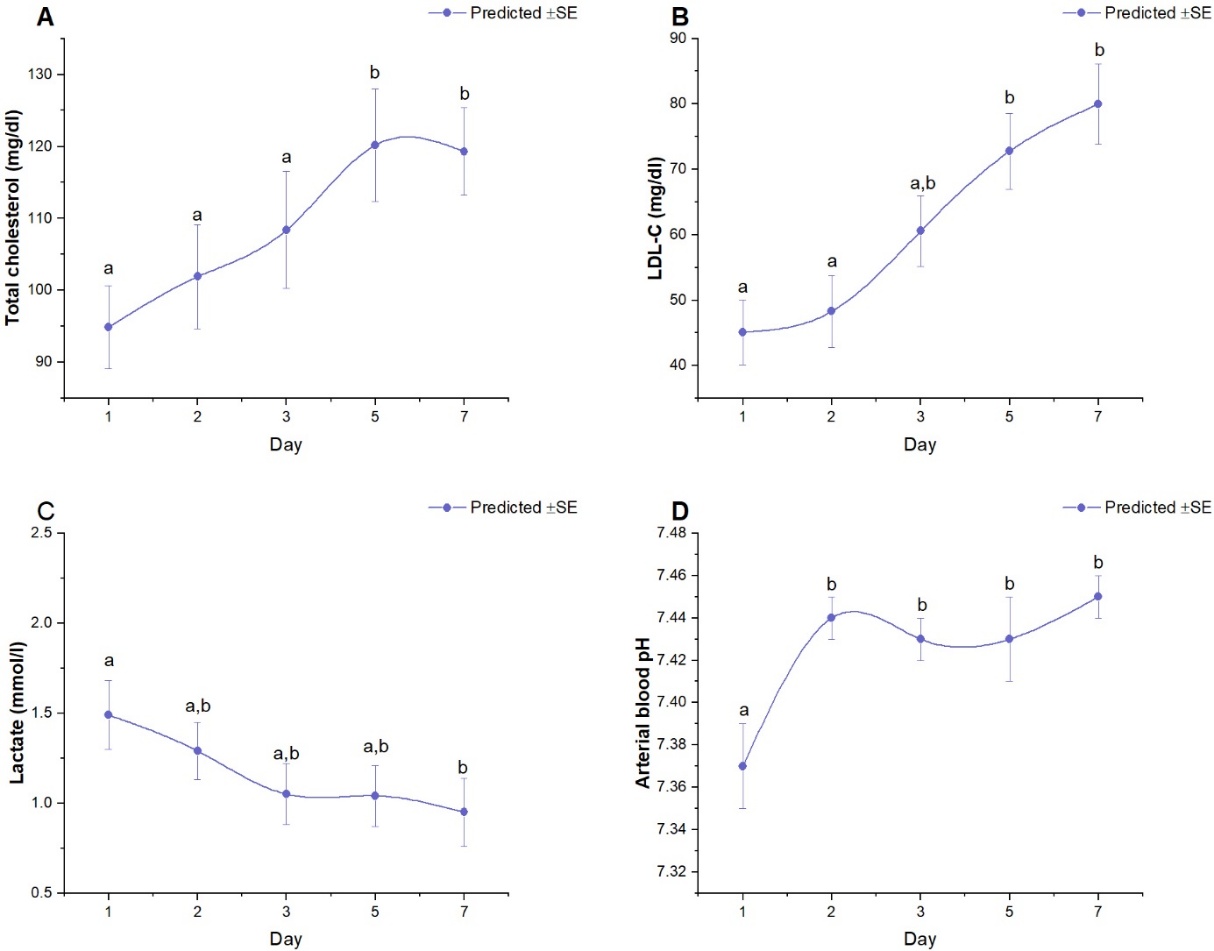


**Figure S4.** Model-based estimates of TC (A), LDL-C (B), lactate (C), and arterial blood pH (D) levels with SE. According to pairwise comparisons, time points not sharing the same letter (a, b) differ significantly in the level of the variable (p < 0.05). Number of patients per time point: Day 1 (n = 30), Day 2 (n = 22), Day 3 (n = 22), Day 5 (n = 18), Day 7 (n = 13).

**Table S6.** Median values (with IQR) and model-based estimates (with standard error) of nutritional parameters over time were calculated using the Generalised Estimating Equations (GEE) model. Statistical significance is provided for comparisons with baseline (Day 1).

| **Variable** | **Measurement** | **N** | **Median** | **IQR** | **Estimated (SE)** | **p-value vs. baseline (Day 1)** |
| --- | --- | --- | --- | --- | --- | --- |
| Energy intake (kcal/day) | Day 1 | 30 | 1249 | 1118–1649 | 1348.2^a^ (119.0) | baseline |
|  | Day 2 | 22 | 1756 | 1253–2107 | 1663.3^a,b^ (108.1) | 0.051 |
|  | Day 3 | 22 | 1925 | 1722–2125 | 1889.0^b^ (90.7) | < 0.001 |
|  | Day 5 | 18 | 1720 | 1538–1974 | 1688.4^a,b^ (87.2) | 0.021 |
|  | Day 7 | 13 | 1565 | 1124–1983 | 1412^a^ (121.0) | 0.801 |
| Protein-to-non-protein intake ratio | Day 1 | 30 | 0.106 | 0.071–0.201 | 0.329^a^ (0.029) | baseline |
|  | Day 2 | 22 | 0.083 | 0.045–0.198 | 0.372^a,b^ (0.030) | 0.307 |
|  | Day 3 | 22 | 0.102 | 0.084–0.202 | 0.409^b^ (0.024) | 0.033 |
|  | Day 5 | 18 | 0.077 | 0.035–0.135 | 0.405^b^ (0.025) | 0.049 |
|  | Day 7 | 12 | 0.076 | 0.061–0.126 | 0.325^a^ (0.044) | 0.942 |
| Non-nutritional energy share | Day 1 | 30 | 0.094 | 0.042–0.199 | 0.356^a^ (0.058) | baseline |
|  | Day 2 | 22 | 0.058 | 0.027–0.101 | 0.221^a,b^ (0.045) | 0.066 |
|  | Day 3 | 22 | 0.038 | 0.011–0.182 | 0.150^b^ (0.025) | 0.001 |
|  | Day 5 | 18 | 0.051 | 0.019–0.178 | 0.118^b^ (0.011) | < 0.001 |
|  | Day 7 | 12 | 0.090 | 0.045–0.187 | 0.162^a,b^ (0.046) | 0.042 |
| REE coverage (%) | Day 1 | 30 | 73.99 | 23.53–121.92 | 70.91^a^ (0.056) | baseline |
|  | Day 2 | 22 | 80.06 | 45.69–145.11 | 84.22^a,b^ (0.056) | 0.094 |
|  | Day 3 | 22 | 86.12 | 46.84–122.52 | 94.79^b^ (0.058) | 0.003 |
|  | Day 5 | 17 | 82.03 | 51.73–120.06 | 82.54^a,b^ (0.047) | 0.113 |
|  | Day 7 | 12 | 68.81 | 32.53–98.93 | 70.35^a^ (0.068) | 0.961 |
| Protein/kg of ABW (g) | Day 1 | 30 | 1.149 | 0.496–1.623 | 1.092^a^ (0.129) | baseline |
|  | Day 2 | 22 | 1.277 | 1.102–1.629 | 1.341^a,b^ (0.122) | 0.154 |
|  | Day 3 | 22 | 1.505 | 1.311–2.063 | 1.623^b^ (0.115) | 0.003 |
|  | Day 5 | 18 | 1.380 | 1.156–1.779 | 1.479^b^ (0.114) | 0.023 |
|  | Day 7 | 13 | 1.372 | 0.923–1.681 | 1.288^a^ (0.127) | 0.875 |
| Protein/kg of IBW (g) | Day 1 | 30 | 1.258 | 0.799–1.702 | 1.216^a^ (0.132) | baseline |
|  | Day 2 | 22 | 1.570 | 1.132–1.911 | 1.521^a,b^ (0.121) | 0.091 |
|  | Day 3 | 22 | 1.989 | 1.504–2.200 | 1.848^b^ (0.102) | < 0.001 |
|  | Day 5 | 18 | 1.673 | 1.432–2.084 | 1.682^b^ (0.117) | 0.009 |
|  | Day 7 | 13 | 1.241 | 0.966–1.709 | 1.239^a^ (0.217) | 0.938 |
| Vitamin A intake (µg/day) | Day 1 | 30 | 1076.1 | 696.6–1339.6 | 1077.7 (115.9) | baseline |
|  | Day 2 | 22 | 1163.1 | 990.0–1469.8 | 1280.2 (102.8) | 0.191 |
|  | Day 3 | 22 | 1251.5 | 1005.0–1612.4 | 1441.0 (114.1) | 0.025 |
|  | Day 5 | 18 | 1224.0 | 930.0–1514.7 | 1379.7 (155.9) | 0.119 |
|  | Day 7 | 13 | 1051.1 | 990.0–1190.0 | 1106.3 (227.1) | 0.912 |
| Vitamin D intake (µg/day) | Day 1 | 30 | 18.22 | 6.15–26.12 | 55.14 (19.83) | baseline |
|  | Day 2 | 22 | 16.66 | 8.61–30.50 | 50.20 (20.90) | 0.741 |
|  | Day 3 | 22 | 28.03 | 15.75–58.21 | 61.79 (21.01) | 0.651 |
|  | Day 5 | 18 | 104.58 | 59.92–119.33 | 72.62 (21.92) | 0.258 |
|  | Day 7 | 13 | 105.02 | 64.88–112.64 | 80.87 (23.21) | 0.117 |
| Vitamin E intake (mg/day) | Day 1 | 30 | 14.85 | 10.84–23.87 | 14.31 (1.57) | baseline |
|  | Day 2 | 22 | 16.22 | 9.11–26.11 | 16.59 (1.47) | 0.278 |
|  | Day 3 | 22 | 19.99 | 10.62–25.54 | 18.97 (1.58) | 0.034 |
|  | Day 5 | 18 | 19.56 | 15.7–28.14 | 19.31 (1.88) | 0.043 |
|  | Day 7 | 13 | 12.14 | 7.65–18.23 | 15.63 (3.33) | 0.736 |
| Vitamin K intake (µg/day) | Day 1 | 30 | 79.75 | 39.02–127.61 | 91.99 (16.78) | baseline |
|  | Day 2 | 22 | 124.51 | 57.24–167.41 | 127.63 (21.21) | 0.190 |
|  | Day 3 | 22 | 99.00 | 74.50–130.01 | 134.57 (32.28) | 0.242 |
|  | Day 5 | 18 | 80.85 | 57.38–101.88 | 96.50 (17.37) | 0.847 |
|  | Day 7 | 13 | 75.90 | 51.36–98.8 | 78.59 (23.34) | 0.643 |
| Vitamin C intake (mg/day) | Day 1 | 30 | 3183.3 | 2667.2–4100.0 | 3576.5 (456.6) | baseline |
|  | Day 2 | 22 | 3167.9 | 2246.9–4120.8 | 3447.4 (708.4) | 0.855 |
|  | Day 3 | 22 | 3132.5 | 2063.9–4174.9 | 3331.9 (700.8) | 0.727 |
|  | Day 5 | 18 | 3100.0 | 1869.6–3486.3 | 2693.4 (713.9) | 0.216 |
|  | Day 7 | 13 | 3100.0 | 1856.0–3428.0 | 2459.1 (744.3) | 0.133 |

IQR – interquartile range; a and b indices show differences between time points according to pairwise comparison.


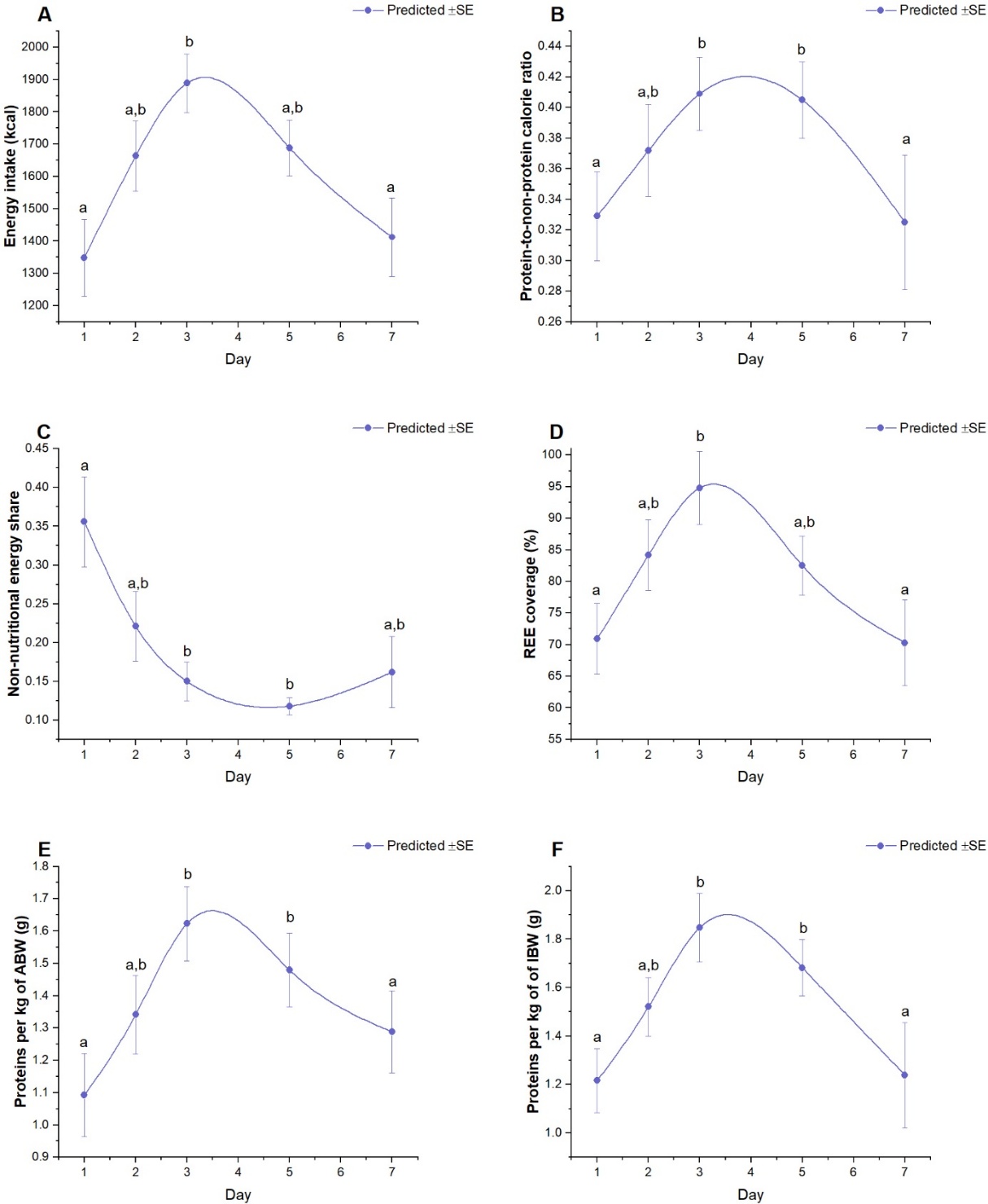


**Figure S5.** Model-based estimates of energy intake (A), protein-to-non-protein calorie ratio (B), non-nutritional energy share (C), REE coverage (D), proteins per kg of ABW (E), and IBW (F) with SE. According to pairwise comparisons, time points not sharing the same letter (a, b) differ significantly in the level of the variable (p < 0.05). Number of patients per time point: Day 1 (n = 30), Day 2 (n = 22), Day 3 (n = 22), Day 5 (n = 18), Day 7 (n = 13).

**Table S7**. Key statistical parameters from separately adjusted linear mixed-effects models assessing the relationship between resting energy expenditure (REE) and clinical variables calculated using linear mixed-effects models.

| **Predictor** | **Slope** | **SE** | **t-value** | **p-value** | **R^2^_marginal_** | **R^2^_conditional_** | **AIC** | **p-value (vs linear)** |
| --- | --- | --- | --- | --- | --- | --- | --- | --- |
| CRRT | -237.09 | 32.6 | -2.131 | 0.036 | 0.251 | 0.713 | 1501 | < 0.001 |
| BMI (kg/m^2^) | 3.200 | 0.742 | 0.273 | 0.787 | 0.279 | 0.696 | 1450 | < 0.001 |
| Temperature (°C) | 136.3 | 29.4 | 4.638 | < 0.001 | 0.372 | 0.663 | 1473 | < 0.001 |
| CRP (mg/l) | -0.391 | 0.046 | -0.917 | 0.362 | 0.211 | 0.723 | 1451 | < 0.001 |
| PCT (ng/ml) | 1.296 | 0.077 | 1.675 | 0.100 | 0.262 | 0.700 | 1503 | < 0.001 |
| IL-6 (pg/ml) | -0.102 | 0.025 | -0.402 | 0.689 | 0.261 | 0.758 | 1172 | < 0.001 |
| WBC (1000/µl) | -1.863 | 0.421 | -0.454 | 0.651 | 0.225 | 0.706 | 1506 | < 0.001 |
| Albumin (g/dl) | 17.02 | 2.91 | 0.453 | 0.652 | 0.217 | 0.707 | 1464 | < 0.001 |
| TC (mg/dl) | 1.376 | 0.486 | 0.926 | 0.357 | 0.228 | 0.720 | 1424 | < 0.001 |
| LDL-C (mg/dl) | 4.792 | 0.618 | 2.961 | 0.004 | 0.287 | 0.771 | 1412 | < 0.001 |
| HDL-C (mg/dl) | -7.261 | 1.136 | -1.414 | 0.161 | 0.242 | 0.697 | 1422 | < 0.001 |
| Triglycerides (mg/dl) | -0.381 | 0.049 | -1.092 | 0.278 | 0.219 | 0.741 | 1450 | < 0.001 |
| Lactate (mmol/l) | -45.145 | 9.361 | -2.332 | 0.023 | 0.270 | 0.669 | 1375 | < 0.001 |
| Glucose (mg/dl) | 0.343 | 0.056 | 0.576 | 0.566 | 0.226 | 0.706 | 1506 | < 0.001 |
| Arterial blood pH | 942.9 | 73.2 | 2.526 | 0.013 | 0.240 | 0.727 | 1500 | < 0.001 |
| Energy intake (kcal/day) | 0.139 | 0.060 | 2.306 | 0.024 | 0.256 | 0.598 | 1431 | < 0.001 |
| Protein-to-non-protein calorie ratio | 972.1 | 34.9 | 2.825 | 0.006 | 0.245 | 0.754 | 1402 | < 0.001 |
| Non-nutritional energy share | -470.4 | 88.5 | -2.369 | 0.021 | 0.259 | 0.662 | 1350 | < 0.001 |
| REE coverage (%) | -187.5 | 46.4 | -1.006 | 0.318 | 0.252 | 0.487 | 1356 | < 0.001 |
| Protein/kg of ABW (g) | 145.2 | 43.6 | 1.737 | 0.087 | 0.247 | 0.671 | 1378 | < 0.001 |
| Protein/kg of IBW (g) | 178.4 | 39.5 | 2.243 | 0.028 | 0.264 | 0.651 | 1377 | < 0.001 |
| Vitamin A intake (µg/day) | 0.001 | 10^-5^ | 0.741 | 0.193 | 0.228 | 0.573 | 1434 | < 0.001 |
| Vitamin D intake (µg/day) | 3.111 | 0.011 | 2.730 | 0.008 | 0.262 | 0.630 | 1420 | < 0.001 |
| Vitamin E intake (mg/day) | 11.12 | 0.69 | 1.517 | 0.134 | 0.252 | 0.641 | 1402 | < 0.001 |
| Vitamin K intake (µg/day) | 1.321 | 0.075 | 1.232 | 0.223 | 0.282 | 0.644 | 1245 | < 0.001 |
| Vitamin C intake (mg/day) | 0.234 | 0.012 | -1.072 | 0.288 | 0.221 | 0.568 | 1437 | < 0.001 |
| SOFA score | -4.869 | 0.6321 | -0.298 | 0.766 | 0.212 | 0.544 | 1425 | < 0.001 |
| APS score | -8.356 | 0.585 | -0.977 | 0.334 | 0.219 | 0.581 | 1426 | < 0.001 |
| RASS score | 58.35 | 3.0166 | 1.934 | 0.057 | 0.240 | 0.593 | 1420 | < 0.001 |
| NUTRIC score | 6.447 | 0.6357 | 0.192 | 0.848 | 0.214 | 0.528 | 1423 | < 0.001 |

SE – standard error, AIC – Akaike information criterion, R^2^_marginal_ – the proportion of variance explained by fixed effects, R^2^_conditional_ - the proportion of variance explained by both fixed and random effects.

**Table S8**. Key statistical parameters from separately adjusted linear mixed-effects models assessing the relationship between the respiratory quotient (RQ) and clinical variables were calculated using linear mixed-effects models.

| **Predictor** | **Slope** | **SE** | **t-value** | **p-value** | **R^2^_marginal_** | **R^2^_conditional_** | **AIC** | **p-value (vs linear)** |
| --- | --- | --- | --- | --- | --- | --- | --- | --- |
| CRRT | 0.021 | 0.003 | -0.750 | 0.456 | 0.103 | 0.210 | 125 | < 0.001 |
| BMI (kg/m^2^) | -10^-4^ | 2.2·10^-5^ | -0.045 | 0.964 | 0.111 | 0.274 | 112 | < 0.001 |
| Temperature (°C) | 0.017 | 0.001 | 1.878 | 0.064 | 0.123 | 0.157 | 124 | < 0.001 |
| CRP (mg/l) | -3.1·10^-5^ | 1.1·10^-6^ | -0.301 | 0.765 | 0.109 | 0.189 | 105 | 0.044 |
| PCT (ng/ml) | 3.4·10^-4^ | 1.9·10^-5^ | -1.789 | 0.077 | 0.124 | 0.218 | 117 | 0.043 |
| IL-6 (pg/ml) | -7.0·10^-5^ | 4.9·10^-6^ | -1.408 | 0.165 | 0.124 | 0.179 | 61 | 0.035 |
| WBC (1000/µl) | -0.002 | 1.1·10^-4^ | -1.598 | 0.141 | 0.134 | 0.199 | 122 | 0.041 |
| Albumin (g/dl) | -0.052 | 0.003 | -1.376 | 0.173 | 0.114 | 0.271 | 119 | 0.043 |
| TC (mg/dl) | -3.5·10^-4^ | 3.2·10^-5^ | -1.066 | 0.291 | 0.078 | 0.406 | 145 | 0.034 |
| LDL-C (mg/dl) | 4.5·10^-4^ | 4.3·10^-5^ | -1.029 | 0.308 | 0.087 | 0.238 | 113 | 0.025 |
| HDL-C (mg/dl) | -1.1·10^-3^ | 1.2·10^-4^ | -0.974 | 0.334 | 0.072 | 0.395 | 147 | 0.041 |
| Triglycerides (mg/dl) | 1.5·10^-4^ | 7.6·10^-6^ | 1.962 | 0.057 | 0.115 | 0.210 | 117 | 0.038 |
| Lactate (mmol/l) | 1.6·10^-3^ | 4.7·10^-5^ | 0.343 | 0.732 | 0.095 | 0.194 | 121 | 0.011 |
| Glucose (mg/dl) | 4.3·10^-4^ | 1.8·10^-5^ | -2.290 | 0.025 | 0.132 | 0.140 | 120 | 0.021 |
| Arterial blood pH | -0.101 | 0.012 | -0.892 | 0.375 | 0.101 | 0.201 | 128 | 0.047 |
| Energy intake (kcal/day) | 4.13·10^-5^ | 6.79·10^-6^ | 2.458 | 0.016 | 0.143 | 0.173 | 115 | 0.044 |
| Protein-to-non-protein calorie ratio | -0.030 | 0.0006 | -0.427 | 0.671 | 0.111 | 0.222 | 124 | 0.042 |
| Non-nutritional energy share | -0.047 | 0.0042 | -1.120 | 0.266 | 0.115 | 0.156 | 124 | 0.041 |
| REE coverage (%) | 0.075 | 0.0033 | 2.240 | 0.028 | 0.138 | 0.210 | 130 | 0.047 |
| Protein/kg of ABW (g) | 0.015 | 0.001 | 0.910 | 0.365 | 0.102 | 0.174 | 124 | 0.044 |
| Protein/kg of IBW (g) | 0.021 | 0.002 | 1.326 | 0.188 | 0.108 | 0.161 | 125 | 0.042 |
| Vitamin A intake (µg/day) | -3.7·10^-6^ | 1.66·10^-7^ | -0.226 | 0.822 | 0.095 | 0.189 | 110 | 0.048 |
| Vitamin D intake (µg/day) | 2.01·10^-5^ | 10^-6^ | 1.084 | 0.203 | 0.110 | 0.228 | 130 | 0.045 |
| Vitamin E intake (mg/day) | 4.1·10^-5^ | 2.1·10^-6^ | 1.255 | 0.112 | 0.166 | 0.631 | 109 | 0.041 |
| Vitamin K intake (µg/day) | 10^-5^ | 10^-7^ | 1.282 | 0.221 | 0.136 | 0.581 | 102 | 0.044 |
| Vitamin C intake (mg/day) | 10^-5^ | 2·10^-6^ | -1.348 | 0.157 | 0.167 | 0.591 | 111 | 0.047 |
| SOFA score | -0.0049 | 0.0003 | -1.401 | 0.166 | 0.120 | 0.187 | 123 | < 0.001 |
| APS score | 0.0024 | 0.0003 | 0.908 | 0.367 | 0.101 | 0.208 | 121 | < 0.001 |
| RASS score | 0.0021 | 0.0002 | 0.250 | 0.803 | 0.095 | 0.192 | 122 | < 0.001 |
| NUTRIC score | 0.0009 | 0.0001 | -1.001 | 0.278 | 0.112 | 0.239 | 123 | < 0.001 |

SE – standard error, AIC – Akaike information criterion, R^2^_marginal_ – the proportion of variance explained by fixed effects, R^2^_conditional_ - the proportion of variance explained by both fixed and random effects.
